# Cavitation bubble interaction with compliant structures on a microscale: A contribution to the understanding of bacterial cell lysis by cavitation treatment

**DOI:** 10.1016/j.ultsonch.2022.106053

**Published:** 2022-06-02

**Authors:** Jure Zevnik, Matevž Dular

**Affiliations:** a University of Ljubljana, Faculty of Mechanical Engineering, Aškerčeva cesta 6, Ljubljana, Slovenia

**Keywords:** Bubble dynamics, Cavitation, Bacteria, Fluid–structure interaction, Water treatment

## Abstract

Numerous studies have already shown that the process of cavitation can be successfully used for water treatment and eradication of bacteria. However, most of the relevant studies are being conducted on a macro scale, so the understanding of the processes at a fundamental level remains poor. In attempt to further elucidate the process of cavitation-assisted water treatment on a scale of a single bubble, the present paper numerically addresses interaction between a collapsing microbubble and a nearby compliant structure, that mechanically and structurally resembles a bacterial cell. A fluid–structure interaction methodology is employed, where compressible multiphase flow is considered and the bacterial cell wall is modeled as a multi-layered shell structure. Simulations are performed for two selected model structures, each resembling the main structural features of Gram-negative and Gram-positive bacterial cell envelopes. The contribution of two independent dimensionless geometric parameters is investigated, namely the bubble-cell distance δ and their size ratio ς. Three characteristic modes of bubble collapse dynamics and four modes of spatiotemporal occurrence of peak local stresses in the bacterial cell membrane are identified throughout the parameter space considered. The former range from the development of a weak and thin jet away from the cell to spherical bubble collapses. The results show that local stresses arising from bubble-induced loads can exceed poration thresholds of cell membranes and that bacterial cell damage could be explained solely by mechanical effects in absence of thermal and chemical ones. Based on this, the damage potential of a single microbubble for bacteria eradication is estimated, showing a higher resistance of the Gram-positive model organism to the nearby bubble collapse. Microstreaming is identified as the primary mechanical mechanism of bacterial cell damage, which in certain cases may be enhanced by the occurrence of shock waves during bubble collapse. The results are also discussed in the scope of bacteria eradication by cavitation treatment on a macro scale, where processes of hydrodynamic and ultrasonic cavitation are being employed.

## Highlights

• Microbubble collapse in vicinity of a bacterial cell is investigated numerically.
• Bubble dynamics ranges from weak jetting away from the cell to a spherical collapse.
• Bubble-induced local stresses can exceed poration thresholds of cell membranes.
• Bacterial cell damage can be explained solely by mechanical effects.
• Microstreaming and shock waves are the primary mechanisms of bacterial cell damage.

## 1 Introduction

Cavitation is a physical phenomenon where changes in ambient pressure cause the formation, growth, and collapse of vaporous and gaseous cavities, commonly known as bubbles. Today, cavitation is being researched far beyond the initial area of interest, which has mainly included the studies in relation to erosion, vibration, and noise in hydraulic machinery. To name a few, cavitation (either ultrasonic or hydrodynamic) can be nowadays encountered in various important applications in the fields of chemistry [1], medicine [2], and for the intensification of chemical and physical processes [3]. Cavitation also poses as a promising new method for water treatment [4], as it has been shown to be able to eradicate bacteria [5], [6], [7], inactivate viruses [8], [9], and destroy other biological structures, such as liposomes [10], [11], [12]. Although cavitation has been studied extensively in relation to water treatment, the understanding of the processes on the most fundamental level remains poor [13]. The latter is especially true when it comes to eradication of bacteria, viruses, and other potentially harmful pathogens on a smaller micro- and nanoscale. Our previous work suggests that during the inertial microbubble collapse the nearby submerged particle can be exposed to shear loads with peaks of a few megapascals and highly variable compressive loads in the form of shock waves with the magnitude in the order of a few hundred megapascals and gradients up to 100 MPa/μm [14]. Additionally, when one considers highly compliant biological structures, like liposomes, a collapsing bubble causes the nearby structure to deform according to the locally induced velocity field, which can lead to local membrane poration or even destruction of the liposome as a whole [15]. Results also suggest that larger bubbles might carry a higher potential for causing stretching-induced liposome destruction, although this has yet to be confirmed experimentally.

Nevertheless, mechanical properties can vary significantly across different potentially harmful pathogens. If we focus on bacteria, numerous bacterial species are recognized as waterborne pathogens and can pose as a threat to human health. Bacterial cells can be found in a variety of shapes, ranging from simple spherical, to more complex rod-shaped, spiral, etc. The typical size of individual cells is in the order of a micrometer and ranges between 0.5 and 5 μm. They can be characterized by a relatively simple cell structure, free of cell nucleus and organelles. When one considers a response of bacterial cells to external loads, bacteria can not be regarded as rigid particles, as they can undergo large deformations and behave like elastic rods when subjected to transient hydrodynamic forces [16]. On the other hand, directly comparing them to simpler biological structures, such as liposomes, could lead to oversimplification of the problem at hand, as bacteria possess a more complex cell envelope consisting of cell membranes and a cell wall, which makes them generally highly resistant to various mechanical loads [17].

To our best knowledge, bubble-bacteria interaction has not yet been studied on the most fundamental level, where a single bubble-bacterium pair is considered. In this light, the present paper numerically addresses an interaction between a collapsing microbubble and a nearby compliant structure that mechanically and structurally resembles a bacterial cell. Thus a here considered phenomenon falls into a broader scope of bubble-structure interaction studies. Modes of single bubble collapse in vicinity of various boundaries and their underlying mechanisms have been a subject of extensive research in the past due to a wide range of practical applications in a range of disciplines [18]. They can vary from spherical collapse, to micro-jet formation towards [19] or away from the boundary [20]. In some cases bubble collapse can also result in a fast thin needle-like jet formation towards the boundary [21] or development of non-axial jets [22]. Additionally, bubbles can also exhibit mushroom-shaped [23] and pear-shaped [24] collapse modes, or even develop multiple opposing jets that lead to bubble splitting and breakup into smaller fragments [25]. In general, bubble collapse dynamics is determined by multiple factors [18], which are related to geometric configuration, material and structural properties of nearby boundaries, and the presence of other jet drives, e.g., shear flow, shock waves, gravity field, or even a presence of other bubbles. .

As many practical and technical difficulties arise during experimental investigations on herein considered spatio-temporal scales, numerical modeling and simulations pose as an important research tool. Perhaps the most widely used approach in the past was to use the boundary integral method (BEM) along with the potential flow theory [26]. Its advantage lies in computational efficiency and ability to consider bubble dynamics in various environments, e.g., vicinity of an elastic fluid [27], elastic membrane [28], and deformable material [29]. In the case of a simultaneous consideration of a nearby deformable solid structure, one can notice a coupled BEM-FEM (finite element method) approach, which allows for more complex cases of structure dynamics. Through the coupled approach, bubble behavior was addressed in vicinity of various elastic and elastoplastic structures [30], [31], [32] and membranes [28], [33], [34], near an elastic gel and within an elastic vessel [35], and besides a submerged sandwich plate [36]. However, a downside to the BEM approach is its limitation to consider linear differential equations, which along with the potential flow theory results in the neglection of viscous effects and compressibility of the surrounding fluid. Although some of these mechanisms normally play a minor role on single bubble dynamics, they gain importance when considering bubble-structure interaction [37], especially on a micro scale [38]. An additional downside to the BEM approach is its limitation to consider simply connected bodies, which requires additional numerical treatment in order to capture bubble jetting, splitting, etc., which are commonly encountered when bubbles collapse in vicinity of various boundaries. A similar downside exists with other interface-tracking methods, e.g., front-tracking method [39]. On the other hand, the same can not be said for interface-capturing methodologies. Numerous numerical studies of single cavitation bubble collapse beyond the point of jet impact or bubble breakup were already performed by either the level-set method [40] or the volume of fluid method [41].

Bubble-structure interaction in compressible liquids has already been addressed by coupling BEM-based solvers with compressible Euler flow solvers. This way, the BEM methodology provides a computationally efficient way of resolving bubble dynamics when velocities are small and compressibility effects can be neglected, while shock wave formation and propagation can be still captured during the bubble collapse and jet impact. Either one-way or two-way FSI was achieved by further coupling with FEM based structural solvers. Models of this type were already successfully used to simulate material erosion and deformation due to a single cavitation bubble collapse [42] and response of a sandwich structure to the nearby underwater explosion bubble [36]. Using a similar methodology, bubble pair and bubble cluster collapse near a deformable material was also considered in the past [43], [44]. While in the recent years two-way FSI studies of bubble collapse near deformable materials were demonstrated by employing purely compressible flow modeling [45], [46], [47], [48], all of the modeling approaches considered inviscid fluids. Although this might be a resonable simplification in various applications, the same can not be said for modeling microbubble-structure dynamics in biomedical and environmental applications [37], such as one considered in the present study.

As already mentioned, in the recent years cavitation has been introduced as a promising method for water treatment and bacteria eradication. Numerous potentially damaging mechanisms that accompany cavitation can be speculated [13]. In general, we can distinguish between mechanical and chemical effects. The former includes strong shear flows [49], jets [50], high local temperatures [51], pressure variability [52], and shock waves [53], whereas chemical effects can be attributed to the high oxidative potential of reactive oxygen species formed during inertial bubble collapse [54]. The contribution of different mechanisms and their possible synergistic effects in various applications are still being explored [13], however the most recent studies that employ the use of low frequency ultrasound [7] and hydrodynamic cavitation [9], [55] for water treatment suggest that mechanical effects are the primary causes for inactivation of various biological structures.

In this light, we presently employ a two-way fluid–structure interaction methodology, which considers the effects of bubble collapse on the bacterial cell and also the mutual effect of cell deformability on the surrounding fluid flow and bubble dynamics. A finite volume method along with the volume of fluid method is used to resolve multiphase flow. This approach has already been shown to successfully resolve various cases of spherical and non-spherical bubble dynamics, such as in vicinity of a rigid wall [56], free surface [57], liquid–liquid interface [58], and in a gravity field [59]. Its advantage lies in a possibility to consider viscous flow and compressibility effects, which is necessary to evaluate shear loads exerted on a nearby compliant structure and to capture shock wave emission and propagation upon bubble collapse. An additional advantage of the volume of fluid method is that it ensures conservation. On the other hand, bacterial cell wall is modeled as a compliant shell structure with multiple layers and hyperelastic material properties. Its dynamic response to the bubble-induced loads is resolved using a nonlinear finite element method based solver.

Following the introduction, a description of the considered problem is presented along with the employed numerical methodology. A brief overview of model setup and preliminary simulations is also included. Results are divided into two main categories. The first part is mainly concerned with bubble dynamics and the resulting loads on a nearby bacterial cell, whereas the second part directly addresses the mechanical response of single bacterial cells to the bubble-induced loads. Following that the presented results are discussed and compared to other relevant studies. In addition, single bubble damage potential for bacterial cell membrane poration is estimated and the results are further discussed in the scope of bacteria eradication by cavitation treatment on a macro scale, where processes of hydrodynamic and ultrasonic cavitation are being employed. Additional details regarding modeling bacterial cells as compliant structures, model setup, and model validation are given in appendices. Supporting video material and extended results are available in supplemental data.

## 2 Methods

### 2.1 Problem Description

Presently we consider an initially stable spherical microbubble with radius $R_{0}$ and in equilibrium with the standard atmospheric ambient pressure of $p_{\infty ,0}=101325$ Pa. As a results, the initial bubble pressure is uniform and amounts to $p_{\infty ,0}+2\gamma /R_{0}$. Here γ denotes surface tension between the bubble and ambient liquid. A sudden ambient pressure increase causes the bubble to collapse violently, causing a highly non-uniform disturbance in the local pressure and velocity field, and subsequently resulting in a gradual deformation of a nearby bacterial cell. A spatially uniform collapse driving pressure of $p_{\infty }=10^{7}$ Pa is considered, which is a typical value one could expect to occur locally in a cavitating flow [60], [53].

**Fig. 1:**
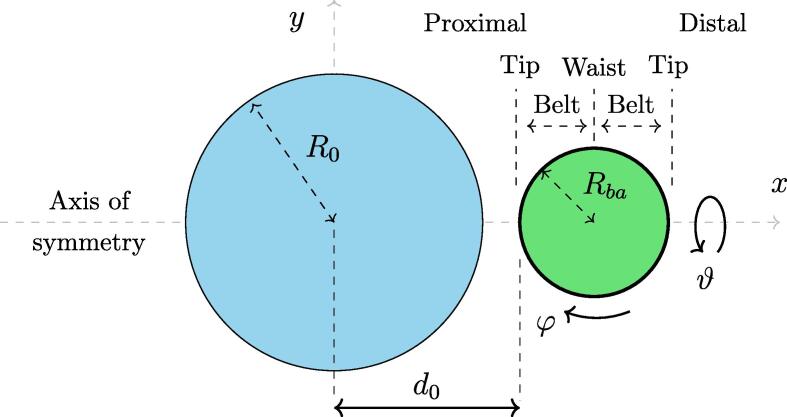
A schematic representation of the considered setup – an initially stable bubble with the initial radius $R_{0}$ (left) in vicinity of a freely submerged spherical bacterial cell with radius $R_{\mathrm{ba}}$ (right).

As the ambient water is assumed to be infinite, only two length scales are present - the initial bubble radius $R_{0}$ and the radius of the bacterial cell $R_{\mathrm{ba}}$. Based on this, any geometrical configuration of the bubble-bacterium pair can be described with two independent dimensionless parameters, their initial distance δ and size ratio ς. They are defined as $\delta =d_{0}/R_{\max}$ and $\varsigma =R_{\max}/R_{\mathrm{ba}}$, where $R_{\max}$ and $d_{0}$ are the maximal bubble radius and the initial distance between the bubble center and the cell wall, as shown in Fig. 1. Additionally, different regions of the cell envelope in relation to the bubble placement are defined: tip, belt, and waist, along with its local coordinates φ and ϑ. The former corresponds to the tangential direction of the envelope in the here considered plane (x-y), whereas ϑ marks the direction of revolution about the axis of symmetry *x*. Non-dimensional distance between bubble and cell center therefore amounts to the sum of δ and 1/ς. Presently bubble collapse and subsequent rebound is considered, therefore $R_{\max}=R_{0}$. For this reason we limit the scope of the initial bubble-bacterium distance to δ>1, although this parameter can generally take values below one when one considers different bubble dynamics scenarios. As bacterial cells are predominantly found on the spatial scale of a micrometer, we decided to keep $R_{\mathrm{ba}}$ constant at 1 μm and change the initial bubble radius instead, which is varied between $R_{0}=0.75$ and 10 μm, as most cavities in water have an initial diameter in the order of a few micrometers [61]. Therefore the bubble-cell size ratio ς corresponds to the initial bubble size relative to the bacterial cell and covers the range between 0.75 and 10, e.g., the value of ς=10 corresponds to the bubble being initially 10-times larger than the bacterium. The third and last variation of model parameters is related to the composition of the bacterial cell envelope. We consider two different model structures, GN and GP structure, where each encapsulates the main mechanical characteristics of the Gram-negative and Gram-positive bacterial cell wall, respectively (see Section 2.3).

### 2.2 Governing Equations of Bubble-Structure Interaction

**Fig. 2:**
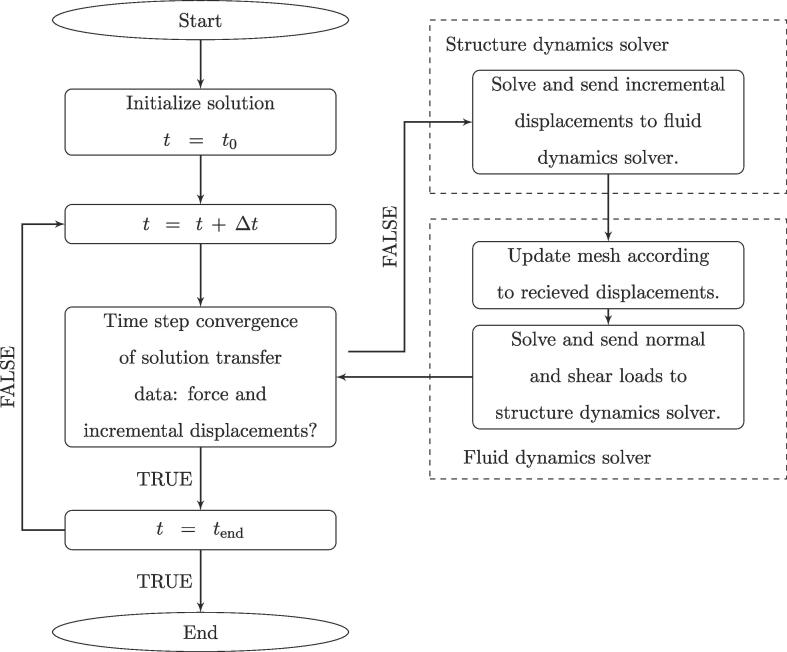
System coupling scheme of the fluid–structure interaction model.

A fluid–structure interaction (FSI) modeling methodology is employed, where the whole domain of interest - a gas bubble, a nearby compliant structure, and ambient liquid, is split into two sub-domains - fluid and structure domain, according to the material characteristics and modeling approaches of each constituent. The dynamic response of each sub-domain is modeled in a separate numerical model - fluid and structure dynamics model, which are coupled together according to the partitioned iterative approach to form the final FSI numerical model [62]. Two-way coupling is achieved trough the exchange of loads (normal and shear forces) and incremental displacements of the FSI interface, which presently comprises the wetted area of the structure. The utilized coupling solution procedure is given in Fig. 2. The presented methodology is largely based on our previous work [14], [15], where a more detailed description of the numerical model can be found.

#### 2.2.1 Fluid Dynamics Model

The presently utilized fluid dynamics solver [63] is based on the finite volume method along with the volume of fluid method to resolve multiphase compressible flow. Generally, bubble dynamics can be mathematically described through the equations of mass, momentum, and energy conservation. In the present case, three fluid phases are considered - gas bubble, ambient liquid, and liquid interior of the structure. As the latter is enclosed within a shell structure, only the bubble-ambient liquid interface needs to be captured. This is achieved by solving continuity equation for the volume fraction field α of the ambient liquid. In the following section, quantities and properties specific to each phase are marked by a corresponding subscript i=g,l,s, which denotes the gas, ambient liquid, and structure’s liquid contents, respectively. Equation of mass conservation for the *i*-th phase can be written as

(1) $$\frac{\partial \left(\alpha_{i}\rho_{i}\right)}{\partial t}+\nabla \cdot \left(\alpha_{i}\rho_{i}V_{i}\right)=0$$

Here, $\rho_{i}$ and $V_{i}$ denote the density and velocity vector field of the *i*-th phase. The volume fraction field $\alpha_{g}$ of the gas phase can be obtained as $\alpha_{g}=1-\alpha_{l}$. After volume fraction fields are known, we can determine the volume-averaged fluid properties ϕ throughout the computational domain surrounding the structure as $\phi =\sum_{i=g,l}\alpha_{i}\phi_{i}$. In the present case this is true for density ρ, dynamic viscosity μ, and thermal conductivity *k*.

Based on the determined material properties, a single momentum (Eq. (2)) and energy (Eq. (3)) equation can be solved, which yields the shared velocity V and temperature T fields.

(2) $$\frac{\partial }{\partial t}(\rho V)+\nabla \cdot \left(\rho V⊗V\right)=-\nabla p+\nabla \cdot \tau +b$$

(3) $$\frac{\partial }{\partial t}(\rho e)+\nabla \cdot \left(V(\rho e+p)\right)=\nabla \cdot \left(k\nabla T\right)$$

Here, *p* denotes pressure, b body forces, τ the viscous stress tensor, and *e* the total specific energy. Heat transfer is only considered between the gas and ambient liquid phase, as preliminary results showed small temperature changes at the FSI interface even when heat flux over the shell structure was neglected (see Section 3.2 for more details). Viscous stress tensor can be for Newtonian liquids expressed as

(4) $$\tau =\mu \left[\left(\nabla V+{\left(\nabla V\right)}^{T}\right)-\frac{2}{3}\left(\nabla \cdot V\right)I\right],$$

whereas the total specific energy is considered as a mass averaged variable

(5) $$e=\frac{\underset{i=g,l}{\sum }\alpha_{i}\rho_{i}e_{i}}{\underset{i=g,l}{\sum }\alpha_{i}\rho_{i}}\text{.}$$

The total specific energy of each phase $e_{i}$ can be expressed as $e_{i}=h_{i}-\frac{p}{\rho_{i}}+\frac{|V{|}^{2}}{2}$, where $h_{i}$ denotes the *i*-th phase specific enthalpy, calculated from the specific heat of that phase and the shared temperature field. The effects of surface tension are only considered at the interface between the gas phase and ambient liquid, as the structure’s interior is enclosed within the solid envelope. The pressure jump across the liquid–gas interface is modeled by a body force term b in the momentum equation according to the Continuum surface force model [64] and can be expressed as

(6) $$b=\gamma \frac{\rho \left(\nabla \cdot \frac{n}{\left|n\right|}\right)\nabla \alpha_{g}}{\frac{1}{2}\left(\rho_{g}+\rho_{l}\right)}\text{.}$$

Here, γ is the surface tension between the liquid and gas phase, whereas n denotes the bubble surface normal, defined as $n=-\nabla \alpha_{g}$.

Both liquid phases are modeled as compressible according to the modified Tait’s equation of state:

(7) $${\left(\frac{\rho }{\rho_{\mathrm{ref}}}\right)}^{n}=1+\frac{n(p-p_{\mathrm{ref}})}{K_{\mathrm{ref}}}\text{.}$$

The term *n* corresponds to the density exponent and $K_{\mathrm{ref}}$ the reference bulk modulus at the reference pressure $p_{\mathrm{ref}}$. The gas phase is modeled by the ideal gas law

(8) $$\rho =\frac{p}{R_{g}^{*}T},$$

where $R_{g}^{*}$ denotes the specific gas constant. The actually considered material characteristics of all three fluid phases are gathered in Table 1. By considering bubble contents as ideal gas, we neglect the fact that cavitation bubbles in general contain a mixture of vapor and noncondensable gases [38]. Through this, we neglect the bubble’s vapor content and its mass transfer mechanisms over the bubble interface. Vapor pressure is small in comparison to the internal bubble pressure during bubble collapse and therefore does not noticeably affect the bubble dynamics in the presently considered cases [14]. However, the mass transfer mechanisms on the other hand could. As the bubble collapses, its contents are compressed, which results in locally elevated temperatures and pressures. In the case of inertial bubble collapse, a fraction of its vapor contents are lost to the ambient liquid through the process of condensation. Even though this does not significantly influence bubble dynamics until the first collapse, the amount of noncondensable gases in the bubble can significantly affect the magnitude of bubble’s rebound and peak bubble temperatures [65]. On the other hand, the diffusion of noncondensable gases has been shown to be negligible on time scales of inertially collapsing bubbles [65]. As exact bubble contents and their adequate consideration in numerical models remains one of the challenges up to this day, we see the use of ideal gas law as a fair approximation for the presently considered phenomenon.

**Table 1:** Considered material characteristics of all three fluid phases. Please refer to Section 2.3 and Appendix A for further explanation and justification of the considered material properties of the structure’s liquid interior.

Phase	Quantity	Value
Ambient liquid - water		
	Reference pressure $p_{\mathrm{ref}}$ [Pa]	101325
	Reference density $\rho_{\mathrm{ref}}$ [kg/$m^{3}$]	998.2
	Reference bulk modulus $K_{\mathrm{ref}}$ [Pa]	$2.2\times 10^{9}$
	Density exponent n [-]	7.15
	Dynamic viscosity μ [Pa s]	$1\times 10^{-3}$
	Thermal conductivity k [W/(m K)]	0.6
	Surface tension γ [N/m]	0.0728
Gas bubble - air		
	Specific gas constant $R_{g}^{*}$ [J/(kg K)]	287
	Dynamic viscosity μ [Pa s]	$1.8\times 10^{-5}$
	Thermal conductivity k [W/(m K)]	0.0242
Structure interior		
	Reference pressure $p_{\mathrm{ref}}$ [Pa]	101325
	Reference density $\rho_{\mathrm{ref}}$ [kg/$m^{3}$]	1100
	Reference bulk modulus $K_{\mathrm{ref}}$ [Pa]	$2.2\times 10^{9}$
	Density exponent n [-]	7.15
	Dynamic viscosity μ [Pa s]	$1\times 10^{-3}$

#### 2.2.2 Structure Dynamics Model

The structure domain is modeled as a compliant shell structure. Its dynamic response to the bubble-induced loads is resolved using a nonlinear finite element method based transient structural solver [66]. The time-varying displacements, strains, and stresses are obtained by solving the following equation of motion

(9) $$M\overset{¨}{u}+C\overset{˙}{u}+Ku=f,$$

where M,C, and K represent the corresponding mass, damping, and stiffness matrices of the structure, respectively. f and u denote the load and nodal displacement vectors, whereas on overdot represents the derivative with respect to time. Large deflections, true stresses, and true strains are considered in the model. The displacement vector u can be obtained from u=x-X, where x and X correspond to the nodal position vectors in the deformed and undeformed state, respectively. From this the deformation gradient tensor F can be obtained as

(10) $$F=I+\frac{\partial u}{\partial X},$$

where I denotes the identity matrix. The deformation gradient is a second-order tensor, which can be decomposed into a product of rotation R and right stretch tensor U. True strain tensor ε is defined as

(11) ε=lnU,

and can be calculated at the locations of the element integration points through the spectral decomposition of U:

(12) $$\varepsilon =\overset{3}{\underset{i=1}{\sum }}\ln\lambda_{i}e_{i}e_{i}^{T},$$

where $\lambda_{i}$ and $e_{i}$ are eigenvalues and eigenvectors of U and thus correspond to the principal stretches and directions, respectively.

For hyperelastic materials there exists an elastic potential function *W*, which is a scalar function of one of the deformation tensors. The derivative of the elastic potential function with respect to the right Cauchy-Green deformation tensor C determines the stress components in the second Piola–Kirchhoff stress tensor S:

(13) $$S=2\frac{\partial W}{\partial C}\text{.}$$

C is defined through the deformation gradient tensor as $C=F^{T}F$ and its eigenvalues correspond to the squares of the principal stretch ratios: $\lambda_{1}^{2},\lambda_{2}^{2}$, and $\lambda_{3}^{2}$. Presently, we utilize the original Yeoh material model [67] for isotropic incompressible hyperelastic materials, where the elastic potential function is defined as

(14) $$W=\overset{3}{\underset{i=1}{\sum }}c_{i}{\left(I_{1}-3\right)}^{i}\text{.}$$

Here, $c_{i}$ are material constants and $I_{1}=\lambda_{1}^{2}+\lambda_{2}^{2}+\lambda_{3}^{2}$ is the first invariant of the right Cauchy-Green deformation tensor. Finally, the true stress tensor σ can be obtained as $\sigma ={\mathrm{FSF}}^{T}$, since incompressible materials are considered and thus det(F)=1.

### 2.3 Modeling Bacteria as Compliant Structures

We are mainly interested in the mechanical properties of bacterial cells and their constituents that significantly contribute to their structural integrity and mechanical resistance on time scales typical for microbubbles (≲1μs). In general, the bacterial cell wall is considered as the main structural component that provides mechanical resistance of single cells [17]. Depending on the composition of the bacterial cell wall we can broadly distinguish between two categories of bacteria: Gram-positive (GP) and Gram-negative (GN) bacteria. The envelope of Gram-negative bacteria consists of a thin peptidoglycan layer between the inner membrane (IM) and the outer cell membrane (OM). Peptidoglycan (PG) is a polymer composed of sugars and amino acids, that form a network structure which gives bacteria shape, transfers turgid loads, and provides structural integrity and protection against mechanical disturbances. Gram-positive bacteria, in contrast, do not have outer membranes, but have a much thicker peptidoglycan layer, making them generally more resistant to mechanical loads [17]. Although there has long been a consensus that the majority of mechanical loads are carried by the peptidoglycan layer in the cell wall [7], the results of recent studies show that other cell building blocks, such as the outer membrane, may also play an important role in maintaining their structural integrity [68]. The interior of a bacterial cell in normal physiological conditions is overpressured in comparison to the ambient liquid, due to the difference in ion concentrations. The intracellular pressure, know as turgor pressure, pushes the inner membrane against the PG layer, which carries the most of the resulting forces in the envelope. Turgor pressure in bacteria has been measured between 0.01 and 0.5 MPa for GN bacteria [69] and as high as 3 MPa for GP bacteria [17]. Experiments on E. *coli* revealed that the PG layer in normal physiological conditions is already under highly stretched state, with areal strains in the order of 50% [69], [68], [70]. Both membranes on the other hand seem to be in a nearly relaxed state [68]. This is consistent with the fact that lipid bilayers and lipid biomembranes rupture at the strain of only a few percent, when subjected to slow loading rates (∼0.1 mN/m/s) [71].

Presently, we omit modeling specific species and strains of bacteria, but rather focus on the main structural attributes that accompany GN and GP bacteria. Therefore a characteristic structure representing a model organism for each group is considered (Table 2). In this way we are able to keep certain attributes between both groups constant, such as cell shape and size, but on the other hand consider different properties of the cell envelope. Through this, we can directly address how a different cell wall structure and stiffness affects the response of a bacterial cell to the nearby collapsing bubble. We consider a spherical cell shape under physiological conditions, which is referred to as a reference ”undeformed” state. The obtained quantities (strains, stresses) are therefore reported in reference to the initial turgid state, if not stated otherwise. Turgor pressure is accounted by considering the actual envelope stiffness under physiological turgid conditions. This is important as the peptidoglycan layer exhibits a high degree of stress-stiffening and presents as much stiffer in turgid state than in its undeformed state [70].

**Table 2:** Considered structural properties of bacterial cells and their cell walls for both Gram-positive and Gram-negative model organisms. Please refer to Appendix A for further explanation and justification of the considered structural properties.

Cell constituent	Property	GN bacteria model structure	GP bacteria model structure
Bacterial cell			
	Shape	spherical	spherical
	Radius $R_{\mathrm{ba}}$ [μm]	1	1
	Turgor pressure [MPa]	0.1	2
Inner membrane			
	Material property	stress softening hyperelastic solid	stress softening hyperelastic solid
	Thickness $\tau_{0}$ [nm]	4	4
	Areal expansion stiffness $k_{A}^{0}$ [N/m]	0.34	0.34
Peptidoglycan layer			
	Material property	stress hardening hyperelastic solid	stress hardening hyperelastic solid
	Thickness $\tau_{0}$ [nm]	2.5	50
	Areal expansion stiffness $k_{A}^{0}$ [N/m]	0.03	0.6
Outer membrane			
	Material property	stress softening hyperelastic solid	-
	Thickness $\tau_{0}$ [nm]	6	-
	Areal expansion stiffness $k_{A}^{0}$ [N/m]	0.525	-

Each constituent of the bacterial cell wall - inner membrane, peptidoglycan layer, and outer membrane, is modeled as incompressible hyperelastic solid according to the Yeoh material model [67]. The considered material characteristics (Table 3) are based on the previous experimental and computational research [70], [72], [71], [68]. Their selection is further explained and justified in Appendix A.

**Table 3:** Considered material characteristics of cell wall constituents - IM, PG, and OM. Please refer to Appendix A for further explanation and justification of the considered material properties.

	Bacterial cell wall layer		
Quantity	Inner membrane	Peptidoglycan	Outer membrane
Density ρ [kg/m^3^]	1100	1300	1100
Reference areal strain ${∊}_{A}^{\mathrm{ref}}$ [-]	0	0.5	0
Yeoh’s hyperelastic material constants [Pa]			
$c_{1}$	$1.33\times 10^{7}$	$2.85\times 10^{6}$	$1.38\times 10^{7}$
$c_{2}$	$-2.38\times 10^{6}$	$1.38\times 10^{7}$	$-2.55\times 10^{6}$
$c_{3}$	$6.28\times 10^{5}$	$2.61\times 10^{7}$	$7.21\times 10^{5}$

### 2.4 Model Setup

Here a brief overview of model setup and preliminary results is given. More details can be found in Appendix B and our previous work [14], [15]. All simulations are performed for an axisymmetric case, as the presently employed methodology in a full 3D approach is well beyond our computational feasibility. The computational domain is represented by a wedge geometry spanning one computational cell in the direction around the axis of symmetry. Boundary conditions at the end of the computational domain were set to wave transmissive pressure outlet with a static pressure of $10^{7}$ Pa, temperature of $20^{\circ }$C, and volume fraction of water $\alpha_{w}$ set to unity. No slip boundary condition is considered at both sides of the cell wall. Although consideration of axial symmetry has been a commonly employed problem reduction tactic with single bubble dynamics studies, it presently still poses as a serious simplification, as it limits the ability to consider a more complex bacterial cell shape and an arbitrary bubble-cell pair placement, or a presence of other driving factors, e.g., confined space, shear flow.

Computational mesh resolution and time step are varied with ς and were selected based on previous [14], [15] and presently conducted mesh and time step independence studies. Our aim was to keep uniform spatio-temporal resolution across ς with mesh spacing of ∼25 cells per minimum bubble radius and a constant time step that corresponds to the Courant-Friedrichs-Lewy condition of $C_{\max}=0.4$. These two conditions were previously shown to result in discretization errors in the order of 5% for both minimum bubble radius and the corresponding maximum pressures in the case of an unbounded microbubble ($R_{0}=1\mu$m) collapse and bubble-liposome interaction ($R_{0}=1\mu$m, δ=1.2, and ς=1). By discretization errors we refer to the difference towards the estimated time-step and mesh-independent solution through the Richardson extrapolation. The reason for different employed mesh resolutions Δx across ς is not only varying initial bubble size $R_{0}$, but also increasing intensity of bubble collapses with larger bubbles. This means that ratios between initial and minimum bubble radii increase with ς, and can be attributed to the varying contributions of viscous and surface tension forces, both of which are scale dependent and are known to have a cushioning effect on bubble collapse intensity. For this reason the finally employed spatio-temporal resolution was chosen based on preliminary simulations of unbounded bubble collapses (see Appendix B). Mesh spacing of the structure domain was chosen in order to satisfy the condition of a conformal mesh at the FSI interface. The reason for this is that bubble dynamics in the present case primarily governs the required numerical resolution. However, for cases with ς⩾5 the resulting structure mesh resolution was doubled, so that it stayed in the order of 300 cells (Δx≈10 nm). The reason for this is the fact that previous simulations of bubble-liposome interaction ($R_{0}=1\mu$m, δ=1.2, and ς=1) [15] showed that the chosen resolution yields discretization errors of peak local principal strains in the order of 0.1%, even when severe local bilayer wrinkling occurs. Nevertheless, mesh independence was additionally checked for both here considered GN and GP model structure at ς=1 and δ=1.01. Both calculated peak stresses in the shell layer that corresponds to the inner cell membrane show discretization errors below 0.1%.

## 3 Results

As performed numerical simulations are relatively demanding in terms of computational resources ($∼10^{4}$ core hours per case), only a fraction of the parameter space δ-ς was evaluated for each of the two model structures. To be more precise, the δ-ς value pairs were chosen as a result of preliminary simulations, such that for each chosen value of 0.75⩽ς⩽10 the corresponding values of δ were spaced around the expected critical distance $\delta^{*}$ for membrane poration and potential cell lysis. Thirty-seven cases are considered it total, with values of δ in the range between 1.01 and 1.15. The actually considered value pairs δ-ς for both model organisms are given in Fig. S1.

### 3.1 Bubble Collapse Dynamics throughout the Considered Parameter Space

Presently encountered bubble collapse dynamics ranges from a formation of a droplet-like shape and a development of a thin axial jet away from the bacterium to spherical collapses and rebounds. From the obtained results we were able to observe and distinguish between three characteristic modes of bubble collapse in the considered δ-ς parameter space:

• mode J - weak jet away from the bacterium,
• mode T - transition between jetting and spherical bubbles,
• mode S - spherical collapse.

**Fig. 3:**
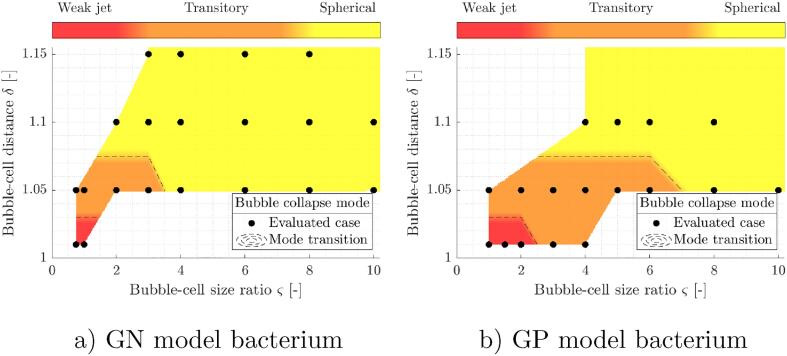
Bubble collapse modes across the considered parameter space δ-ς. Results are given for bubble collapse in vicinity of both considered model structures: a) GN and b) GP model bacterium. The collapse mode transitions (dashed lines) are estimated by interpolation between the numerically evaluated cases (black dots).

Bubble collapse modes across the considered parameter space δ-ς are given in Fig. 3. From here, one can notice that bubble collapse modes tend from J to S with increased values of δ and ς. This is to be expected, as with larger bubble-bacterium distances and size ratios the nearby cell presence yields a bubble environment of lesser anisotropy. A comparison between both the GN and GP cases shows similar bounds between bubble collapse mode transitions along the parameter δ, however the estimated boundaries do vary along the bubble-bacterium size ratio. As was initially expected, bubble shape deviations and collapse anisotropy is generally more pronounced with GP bacteria. This can be explained by higher stiffness of the GP model structure, which presents a greater disturbance for a nearby collapsing bubble. Here it is important to acknowledge that these results do not exclude the possible occurrence of other bubble collapse modes, e.g., bubble jetting towards the bacterial cell, when other bubble-cell geometric configurations are considered, with different material and structural cell properties, and also in the presence of other jet drives.

**Fig. 4:**
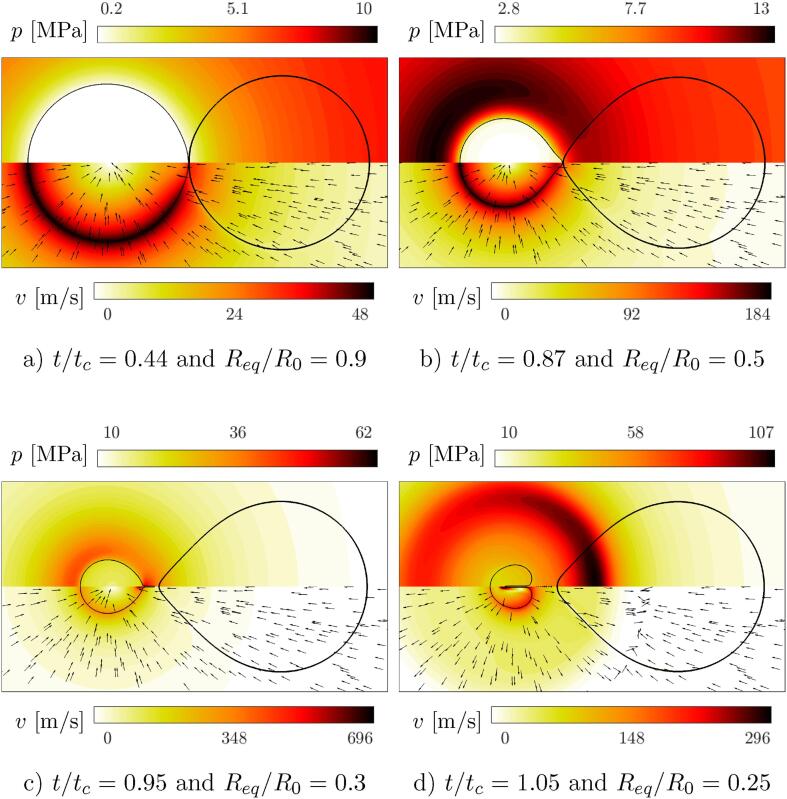
A sample case that resembles bubble collapse mode J - weak jet away from the bacterium: GP model structure, ς=1, and δ=1.01. The corresponding bubble collapse time is $t_{c}=9.98$ ns and the corresponding minimum equivalent bubble radius is $R_{\mathrm{eq}}=116$ nm. The peak bubble rebound is observed at $t/t_{c}=1.53$, with $R_{\mathrm{eq}}/R_{0}=0.52$. Bacterial cell is positioned on the right-hand side of the bubble and the shapes of both are marked by a solid black line. The arrows in the lower half indicate only the direction of the velocity vector field. A corresponding video file showing bubble and bacterium shape progression is available in supplementary material (Video S2).

The bubble collapse modes were primarily identified and differentiated by visualization of bubble shape development. For each of the three identified bubble collapse modes one sample case is selected and a corresponding video file showing bubble and bacterium shape progression is available in supplemental data (Videos S2-S4). Additionally, the temporal development of bubble-bacterium shape and the corresponding pressure (upper half) and velocity (lower half) field contours are given for all three sample cases (Figs. 4, S5, and S6). However, only the one that resembles bubble collapse mode J (GP model structure, ς=1,δ=1.01) is presented more thoroughly in the following pages.

The main features of the identified bubble collapse mode J are a formation of a droplet-like shape, which is followed by a development of a thin uniaxial jet away from the cell. The jet continues to propagate through the bubble during the rebound phase and can be characterized as weak. Mode J is observed only for evaluated cases with δ=1.01 and ς≤2, when the bubble is initially spherical and almost in a direct contact with the cell wall. Due to the ambient pressure increase the bubble begins to contract (Fig. 4 and a characteristic sink-type flow field is induced in the ambient liquid. The latter decays approximately with the square of the distance away from the bubble wall, which results in spatially highly variable flow field development along the bacterial envelope and drives cell deformation and displacement towards the bubble. From velocity field contours we can clearly see the developed boundary layer along the cell wall, which causes the proximal region of the bubble to remain in direct vicinity of the cell wall and lag behind in comparison to the rest. With time, this results in bubble shape deviation from the initial spherical to the formation of a droplet-like shape, with the tip towards the bacterium (Fig. 4b). At this stage, the proximal side of the cell wall has already undergone large deformations, which can be primarily attributed to the mechanism of microstreaming.

As the bubble contracts to the half of its initial size ($R_{\mathrm{eq}}/R_{0}=0.5$ at $t/t_{c}=0.87$), a clear pressure field non-uniformity is present in the ambient liquid at the distal side of the bubble, with its peak (13.1 MPa) already surpassing the collapse driving pressure of 10 MPa. With peak intracellular pressure of 11.6 MPa at the proximal tip, a pressure field gradient of ≈0.85 MPa/μm exists across the bubble wall along the axial direction. Based on this, one might expect that the bubble will collapse towards the denser cell ($\rho_{\mathrm{cell}}/\rho_{\mathrm{water}}=1.1$). However, during the final stages of the collapse ($t/t_{c}=0.95$ and $R_{\mathrm{eq}}/R_{0}=0.3$) the non-uniformity in bubble shape increases and the bubble wall detaches and accelerates away from the bacterium, as the cell envelope is now elongated and thus significantly stiffer than in the initial undeformed state. The bubble collapse anisotropy is thus primarily governed by two opposing factors, the presence of a nearby denser liquid (cell interior) and a compliant shell structure (cell wall).

Following detachment, shear flow along the cell wall drives the formation of a needle-like bubble tip (Fig. 4c), which is later impinged and a small part of the bubble breaks off. As can be seen on the pressure and velocity contours the tip impingement and break-off is accompanied by high local pressures and velocities, surpassing 50 MPa and 500 m/s as the individual bubble fragments collapse. Tip impingement is followed by the development of a thin axial jet away from the bacterium, which continues to propagate through the bubble during the bubble collapse and subsequent rebound (Fig. 4d). During the bubble collapse the pressure and temperature within the bubble reach the peaks of 1.4 GPa and 3500 K. However, these are short lived and spatially limited only to the center of the collapsing bubble. It would be therefore incorrect to consider these values as the actual loads on a nearby cell (see Section 3.2). As the bubble rebounds, the jet is enclosed by an expanding bubble wall, which results in small water droplets being encloses within the bubble. A shock wave is emitted and the shock front shows higher values towards the denser bacterium. The pressure wave decays approximately with the inverse of the distance away from the bubble center, which is the reason for the peak of 205 MPa at the time when shock front reaches the cell wall. The latter value thus represents almost a sevenfold decrease in comparison to the peak calculated internal bubble pressure of 1.4 GPa. Along with the shock front, velocity field abruptly changes its direction and the flow field is reversed to the source-type, which also causes the reversal of the cell’s mode of deformation. No reflection of the shock wave is observed when it propagates past the cell wall and the cell itself, which is due to a negligible difference in acoustic impedance between the bacterial cell and ambient water.

The jet remains within the bubble throughout the rebound and finally manages to pierce the bubble when the maximum rebound bubble size is reached. In this manner, we characterize the developed jet as weak, as it only manages to pierce the bubble during the rebound phase. Although bubble dynamics is qualitatively similar between the cases (mode J), variations can be observed in the length of the formed tip and the size of the impinged part of the bubble. Weak jets are developed in all the evaluated cases classified under bubble collpase mode J, however their strength varies as in some cases the jet does not pierce the bubble even during the bubble rebound phase. Overall, the anisotropy of the bubble environment is not great enough to cause the development of strong or intermediate jets, which pierce the bubble early in the collapse phase or close to the actual collapse and can be then clearly seen during the bubble rebound phase [73].

Bubble collapse mode T can be seen as a transition between jetting (mode J) and spherical (mode S) bubbles, as they still show features similar to the ones observed in mode J. However, the main difference lies in the lack of clear jet development away from the bacterial cell. Mode T is identified for eleven evaluated cases, most of them with δ=1.05 when the bubble is initially in the direct proximity of the cell. Bubble shape deviation away from the initial spherical is less apparent and can be only observed in the late stages of the collapse, when the proximal bubble wall accelerates away from the cell wall. This could be seen as an initial stage of the axial jet development away from the bacterium. The developing jet, however, is cushioned during the collapse and does not get enclosed by the expanding bubble during the rebound, when the bubble again assumes almost spherical shape.

As bubble-bacterium distance and size ratios increase the bubble shape deviations from the initial spherical become less and less evident. Bubbles identified with the collapse mode S remain spherical during the collapse and a subsequent rebound. For bubbles in this category, the effect of a nearby bacterium on the bubble dynamics can be regarded as negligible.

### 3.2 Loads Exerted on a Bacterial Cell

When considering mechanical loads we can distinguish between locally induced loads or loads exerted on a bacterial cell as a whole. We briefly consider both, the former by addressing peak local pressures and shear stresses exerted locally on a cell wall and the latter by looking at the net hydrodynamic force acting on a cell wall as a whole. Key results are gathered in Table 4.

**Table 4:** Peak loads exerted on a bacterial cell during a single microbubble collapse.

Peak load	Model cell	Value interval	Temporal incidence	Spatial incidence	Driving mechanism	Trend along	
						δ[-]	ς[-]
Force [μN]	GN	[2.4,6.7]	$1.03<t/t_{c}<1.05$	Cell wall	Shock wave	Decreasing	Non-monotonic, peak at ς=6
	GP	[10.1,22.1]					Increasing, plateau at ς⩾8
Shear stress [MPa]	GN	[0.56,5.0]	$0.9<t/t_{c}<1.05$	Proximal belt	Bubble detachment or shock wave	Decreasing	Decreasing
	GP	[1.5,4.7]	$1.02<t/t_{c}<1.04$		Shock wave		
Pressure [MPa]	GN	[151,223]	$1.02<t/t_{c}<1.04$	Proximal tip	Shock wave	Decreasing	Increasing
	GP	[166,220]					

First, we comment the peak local loads in the form of maximum calculated pressures at the cell wall. They occur at the time when shock wave reaches the cell and assume values between 151 and 223 MPa. Values decrease with higher δ and increase with ς, which is a result of stronger collapses of larger bubbles where surface tension and viscosity have a lesser cushioning effect on bubble collapse strength. The values are also higher for GN model cell as its vicinity results in bubble dynamics of lesser anisotropy in comparison to the GP model structure and thus in higher magnitudes of emitted shock waves. Peak local shear stresses are also tightly related to the shock wave propagation. Presently the values range between 0.56 and 5 MPa and decrease with both δ and ς. This suggests that smaller bubbles exert higher local shear stresses on nearby cell envelopes and could be explained by a more localized effect of smaller bubbles on nearby cells, which is inherently linked to the geometry of the bubble-cell setup. Additionally, peak shear stresses are higher for GP model bacterium, which is consistent with more evident boundary layer development along the wall of GP model structures (see velocity field contours in Figs. 4a and S5a). Here two exceptions exist (GN, δ=1.01,ς⩽1), where peak shear loads (∼5 MPa) occur already at the time of bubble detachment from the proximal cell tip, as they are driven by shear flow resulting from bubble wall acceleration away from the cell.

As axisymmetric cases are considered, the resultant hydrodynamic force acting on a cell wall only has an axial component. For all the evaluated cases peak resultant forces occur in the direction away from the bubble and are a result of shock wave propagation through the cell. Time of occurrence ranges between $1.03<t/t_{c}<1.05$ and is dependent on the bubble-cell distance, as with larger values of δ the shock front has to travel a longer distance to reach the cell. Peak hydrodynamic forces range between 2.4 and 6.7 μN and between 10.1 and 22.1 μN for GN and GP model bacterium, respectively. For the latter, the values are consistently higher in comparison to the former model structure, as was initially expected due to different cell stiffness. Values of peak net forces are also consistently higher with lower bubble-cell distances, however the trend is not so clear for bubble-bacterium size ratio ς, where an initial steep increase is followed by a plateau or even a decrease with increasing values of ς beyond 6 and 8 for GN and GP model structure, respectively. The magnitudes of peak hydrodynamic forces are thus in the range of few to a few tenths of micronewton, which is relatively high. For reference, during motility through water bacteria experience drag forces in the order of ∼0.1 to 1 pN. Furthermore, when bacterial cells are locally probed using atomic force microscopy, the forces are usually in the order of a nanonewton [69], [68]. However, one does have to keep in mind that these are locally applied on a small fraction of the bacterial cell wall in contrast to the here reported hydrodynamic forces, that act on a cell as a whole. Additionally, loading time and frequency is also an important factor to consider. Shock wave induced loads are highly transient, with relatively short loading times in the order of a few nanoseconds. During that time the resulting hydrodynamic force also abruptly changes its direction from outward (away from the bubble) to inward action (towards the bubble) as determined by the local pressure gradient. On the other hand, hydrodynamic forces exerted on a cell wall during bubble contraction and later during rebound are one to two orders of magnitude lower in comparison to the overall peak force. However, they can be characterized by a longer time of action, which is dependent on the bubble collapse time. Overall, the temporal progression of hydrodynamic loads (net force, local shear stresses and pressures) is very similar to the ones obtained in our previous study, which addressed microbubble dynamics in vicinity of a solid particle of a similar size. For this reason, interested readers are further referred to [14]. Rather than with bubble-induced loads we are presently more interested in the response of a bacterial cell to these loads. This includes cell deformations and local stresses in the cell wall, which are more thoroughly addressed in the following subsection.

Additionally, one has to consider the possibility of cell damage by thermal loads. Although bubble contents can reach upwards of a few thousand Kelvin during the final stages of collapse, here obtained local temperature increase at the cell wall is in the order of a few Kelvin, which is not enough to cause temperature-dependent cell damage. The reason for this is, that highly elevated temperatures are only limited to the bubble interior, as the thermal boundary layer is thin in comparison to the initial bubble size and presently considered bubbles are not attached to the cell wall during their collapse. However, we do acknowledge that the bubble interior was modeled as air obeying the ideal gas law and without the consideration of mass transfer mechanisms, which could affect the thickness of the developed thermal boundary layer. Further studies are thus needed to draw more concrete conclusions.

### 3.3 Bacterial Cell Response to the Bubble-Induced Loads

#### 3.3.1 Modes of Cell Deformation

Initially spherical cells are subjected to bubble-induced loads, which leads to cell translation and shape deformation. A collapsing bubble locally induces a sink type pressure and velocity field in the surrounding liquid. During the final stages of bubble collapse a shock wave is emitted and the bubble rebounds. When the shock wave propagates through the ambient liquid, flow field is suddenly reversed to the source type as the bubble has already begun to expand. Local flow and pressure field development is an important factor as it determines the mode of initial cell deformation. For a simplified case of a spherical bubble in an incompressible liquid the ambient velocity field has only a radial component and can be written as

(15) $$V(r,\theta ,\varphi ,t)=\left[\overset{˙}{R}R^{2}r^{-2},0,0\right]\text{.}$$

Here, r,θ,φ denote spherical coordinates with the origin in the center of the bubble. In this case, the flow is irrotational and the corresponding rate of strain tensor is

(16) $$e=\left[\begin{array}{ccc} -2\overset{˙}{R}R^{2}r^{-3} & 0 & 0\\ 0 & \overset{˙}{R}R^{2}r^{-3} & 0\\ 0 & 0 & \overset{˙}{R}R^{2}r^{-3} \end{array}\right],$$

which suggest that shear rate is zero and only normal strain rates are present in the flow field. From this, we can expect that for a spherically collapsing bubble ($\overset{˙}{R}<0$), a sufficiently small and incompressible fluid parcel will undergo translation towards the bubble, elongate in the radial direction and contract in the corresponding polar and azimuthal directions by one half of the magnitude of elongation. On the other hand, in the case of bubble expansion ($\overset{˙}{R}>0$) the fluid parcel will contract in the radial direction and elongate in polar and azimuthal directions in order to satisfy volume conservation. Although this is only true for a fluid parcel with no inherent elasticity, it still corresponds well with the herein observed modes of bacterial cell deformation. This should come as no surprise, as cells generally show a high level of compliance. At this point, the reader is referred back to SubSection 3.1 and supplementary video files of bubble and bacterium shape progression. From there one can observe a characteristic cell response, which is true for all the evaluated cases. The bacterium is initially elongated and contracts perpendicularly to the axis of elongation. In other words, the distance between both tips increases and the circumference of the waist decreases. Depending on the size ratio to the nearby bubble, the cell also undergoes a certain level of translation towards the collapsing bubble.

**Fig. 5:**
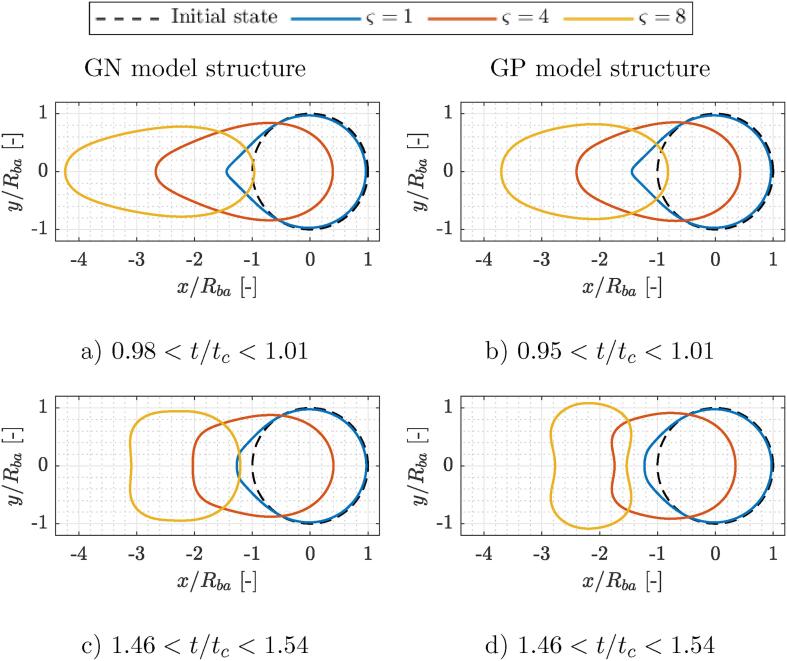
Contours of GN (left column) and GP (right column) model bacterium cell walls at the time of peak cell elongation (a,b) and maximum bubble size during rebound (c,d) in comparison to the initial cell shape and location (dashed line). Contours are given for three values of bubble-bacterium size ratio ς=1,4,and8 and correspond to the cases with δ=1.05. The bubble is located on the left side of the cell for all the cases, but is not shown for the sake of better figure readability. The exact time instants of all shown configurations are given in supplemental data (Tabs. S1 and S2).

Peak cell elongation generally coincides with the bubble collapse. For all the evaluated cases it occurs between $0.94<t/t_{c}<1.01$. GN model structure consistently shows peaks at later times in comparison to the GP structure. For both model structures the peak cell elongation occurs earlier with larger bubble-bacterium size ratios and is higher with smaller values of δ, when bacteria are placed closer to the bubble. Contours of both model cell shapes at the time of peak cell elongation are shown in top row of Fig. 5. One can notice that cell deformation modes at the time of bubble collapse are qualitatively similar between both model organisms (Fig. 5, Fig. 5), however there exist significant differences across the parameter ς, which suggests that bubble-cell size ratio plays an important role on the mode of overall cell deformation. For bubbles of similar size to the bacterium (ς⩽1.5) the distal pole of the cell is barely displaced, the proximal side of the bacterium, however, undergoes a visible elongation towards the collapsing bubble (see videos in supplementary material). With larger bubbles cell deformation gradually becomes more uniform and initially spherical cells temporary assume a capsule-like shape (see Fig. 5, Fig. 5, case ς=8). Additionally, at increased values of ς the magnitude of cell translation intensifies greatly, as at ς=8 the distal tip already undergoes translation of a similar magnitude to the initial cell size ($2R_{\mathrm{ba}}$).

Following the bubble collapse and shock wave propagation past the cell, a source type flow begins to drive cell deformation. The distance between both tips reduces and waist circumference increases. This can be clearly seen in Fig. 5, Fig. 5, where contours of GN (left column) and GP (right column) model bacterium cell walls at the time of maximum bubble size during rebound in comparison to the initial cell shape and location (dashed line) are shown. Again, similarities can be observed between the shapes of both model organisms, however, the GP model structure shows more pronounced shape deformations, which also increase with ς. A detailed observation (see Fig. 5, Fig. 5, case ς=8) reveals cell contraction along the axis of symmetry (abscissa) beyond the initial size of $2R_{\mathrm{ba}}$. Similarly, circumference of the cell waist extends towards the initial value and for the GP model structure even beyond that ($y/R_{\mathrm{ba}}>1$). Both occurrences suggest a significant elastic response of the cell wall, which tends to be greater for GP model bacterium. This is to be expected, as the presently considered GP model structure is roughly two times as rigid as the GN one.

**Fig. 6:**
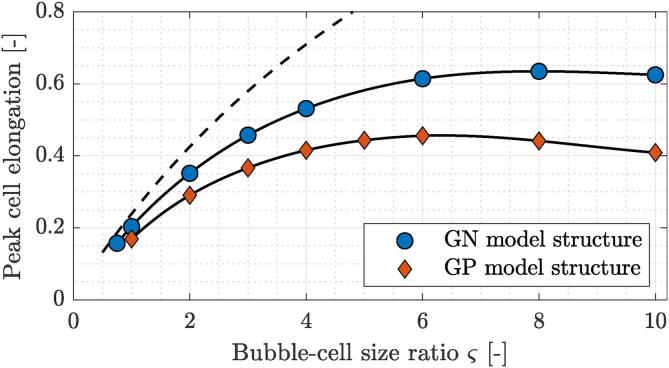
Peak cell elongation in relation to bubble-bacterium size ratio ς for Gram-negative and Gram-positive model organisms at δ=1.05. Dashed line denotes the estimated peak elongation of an equivalent fluid parcel with no inherent elasticity.

A significant elastic response of the cell wall can be also observed when comparing the magnitudes of peak cell elongation in Fig. 6. Results correspond to both model structures at δ=1.05. Presently, cell elongation is defined as engineering strain of cell length, or in other words, as a fraction of the increase in the distance between both cell tips relative to the initial state ($2R_{\mathrm{ba}}$). According to the complementary simulations in our previous study [14], the peak elongation of an inelastic fluid parcel due to the nearby bubble collapse is a strictly increasing function of their size ratio ς and the inverse of their initial distance $\delta^{-1}$. In the present case, however, we can observe a clear trend with a steep initial increase and a local maximum at ς=8 and ς=6 for the GN and GP model organism, respectively. The magnitude of peak cell elongation across the parameter space ranges between 0.15 and 0.64 for the GN model bacterium and between 0.17 and 0.46 for the GP model bacterium. Again, a comparison between both curves reveals a stiffer response of the GP model structure, as it consistently shows smaller peak cell elongations in comparison to the GN model structure.

#### 3.3.2 Local Stress Field in the Inner Cell Membrane

**Fig. 7:**
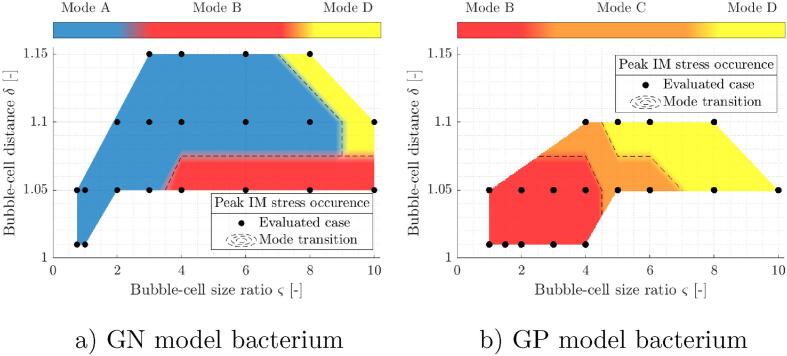
Peak IM stress occurrence modes across the considered parameter space δ-ς. Results are given for bubble collapse in vicinity of both considered model structures: a) GN and b) GP model bacterium. Black circles denote the numerically evaluated cases and black dashed lines the estimated mode transitions.

Rather than considering the overall stress field progression in the bacterial cell envelope, we decided to primarily focus on the temporal and spatial incidence of peak local stresses in the inner cell membrane. This way, we are able do distinguish between different characteristic responses of cells to the nearby collapsing bubble at conditions that result in inner membrane stresses close to the considered poration threshold of 20 MPa (see Appendix A). Overall, we were able to identify four characteristic modes of spatial and temporal incidence of peak local stresses in the inner cell membrane. They are presented in Table 5 and are further attributed to the simulation parameters δ and ς in Fig. 7. In the table, $\sigma_{11}$ and $\sigma_{22}$ denote the stresses in both principal directions of the inner cell membrane. Both principal directions correspond to local cell wall coordinates φ and ϑ (see Fig. 1). For all identified modes, the time of peak stress occurrence increases with parameter δ and decreases with ς. All four modes primarily result from the in-plane extension of the membrane. The only exception to this is in mode B, where $\sigma_{22}$ increases with ς and cases with lower ς show compressive stresses in the second principal direction. For all four identified modes the contribution of local membrane bending to the stress state is in the order of 0.1% and thus we see it as negligible in the present case. Again, here we refer to the inner cell membrane in terms of common biological terminology and not as a two-dimensional structural element with negligible bending stiffness. However, based on the presented results, one could justify the consideration of cell membranes as structural membranes in future modeling endeavors.

**Table 5:** Identified characteristic modes of spatial and temporal incidence of peak local stresses in the inner cell membrane.

Mode label	Stress mode	Spatial incidence	Temporal incidence
A	Equibiaxial tension	Proximal tip	Bubble expansion during rebound
	$\sigma_{11}\approx \sigma_{22}>0$	φ≈0	$1.05<t/t_{c}<1.31$
B	In-plane tension/compression	Proximal belt	Bubble collapse
	$\sigma_{11}>\sigma_{22}$ and $\sigma_{11}>0$	$0<\varphi <\frac{\pi }{2}$	$0.93<t/t_{c}<1.00$
C	In-plane tension	Distal belt	Following shock wave propagation
	$\sigma_{11}>\sigma_{22}>0$	$\frac{\pi }{2}<\varphi <\pi$	$1.07<t/t_{c}<1.14$
D	Equibiaxial tension	Distal tip	Following shock wave propagation
	$\sigma_{11}\approx \sigma_{22}>0$	φ≈π	$1.05<t/t_{c}<1.10$

Temporal incidence of a certain mode does not differ between both model organisms and the four identified modes are not exclusive for a given set of parameters (cell type, δ,ς), as multiple can occur for a single case. However the magnitude of their expression does change across the parameter space, e.g. in the case corresponding to the GP model organism at δ=1.05 and ς=2 the mode B is predominant and represents the overall maximum, however mode A also occurs as a local maximum. In GN model organism the overall peak stresses in inner cell membrane occur correspondingly to modes A, B, and D. On the other hand, for GP model structure the following critical modes were identified: B, C, and D, as can be seen in Fig. 7. Transitions between identified modes of peak stress occurrence in the inner cell membrane are denoted by a black dashed line. They were obtained by linear interpolation between the evaluated cases and only serve as an estimation of the actual mode transitions.

The results show that the composition and rigidity of the cell envelope does affect the mode of its response and consequently the location of potential membrane poration or even cell destruction. This also holds when the nearby bubble dynamics is qualitatively similar, e.g. at δ=1.05 and ς=10 both the GN and GP case result in spherical bubble dynamics (see Fig. 3), however the location of peak stresses in the inner cell membrane varies between the proximal belt (mode B) and distal pole (mode D) for the GN and GP model organism, respectively. We can also check whether there exists a relation between the previously identified bubble collapse modes (see SubSection 3.1) and here shown expression of the peak stresses in the inner cell membrane. A visual comparison of Fig. 3, Fig. 7 reveals some similarities in identified mode transitions, however they are far from identical. For example, when a GN model organism is considered at δ=1.05, the transition between transitory bubble collapse (mode T) and spherical collapse (mode S) occurs at 3<ς<4, which coincides with a transition between stress modes A and B. Similarly, for a GP model organism at δ=1.05, both bubble collapse and stress mode transition occurs at 6<ς<8. This leads us to believe that there might exist a mutual connection between the bubble collapse mode and the resulting occurrence of peak stresses in the cell wall even in the present case, where bubbles are generally in an environment of relatively low anisotropy in comparison to other well known cases, e.g. bubble collapse in vicinity of a rigid wall.

**Fig. 8:**
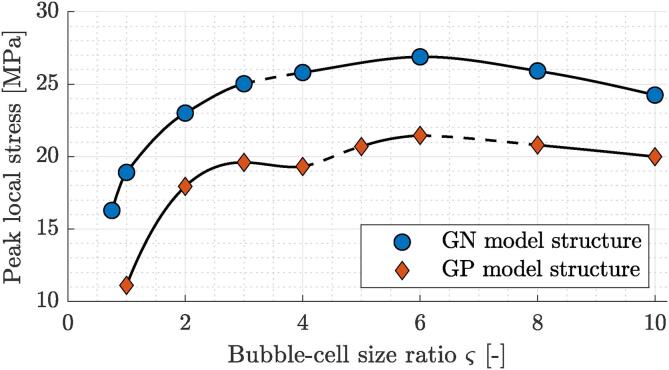
Peak local stresses in the inner cell membrane in relation to bubble-bacterium size ratio ς for Gram-negative and Gram-positive model organisms at δ=1.05. Dashed line denotes the transition between the spatial and temporal incidence of peak local stresses in the inner cell membrane.

Additionally, we also quantitatively compare peak local stresses in the inner cell membrane in relation to bubble-bacterium size ratio ς. They are shown in Fig. 8 for both model organisms at δ=1.05. Here, a dashed line denotes the transition between the spatial and temporal incidence of peak local stresses in the inner cell membrane. Results clearly show that peak membrane stresses in both GN and GP bacteria can exceed poration thresholds of cell membranes, however GP model structure consistently shows lower peaks and thus higher resistance to bubble-induced cell damage. Both curves show non-monotonic trends with maxima at ς=6. For GP model organism there also exists a local maximum at ς=3. The non-monotonic trend can be partially explained by the existence of different modes of peak stress occurrence across the considered parameter space, however they also hint towards a possible presence of a resonant cell response. Based on the modal analysis of both model cell envelopes, we estimate the upper boundary of first natural frequencies of both model organisms to be between 15 and 30 MHz. This corresponds well with location of the peak stress response at ς=6, for which the bubble collapse rate ($1/t_{c}$) equals 17.1 MHz. As we consider only one collapse and the subsequent rebound, the bubble collapse rate might be a misleading metric. Thus, it is given only for the sake of direct comparison with the natural frequencies of both model organisms. Also, this result currently only serves as a speculation and will be further investigated in the future by decoupling the effects of geometric parameters (δ,ς) and bubble collapse frequency, which is primarily determined by the initial bubble radius and the collapse driving pressure.

## 4 Discussion

### 4.1 Estimation of Single Bubble Damage Potential for Bacteria Eradication

**Fig. 9:**
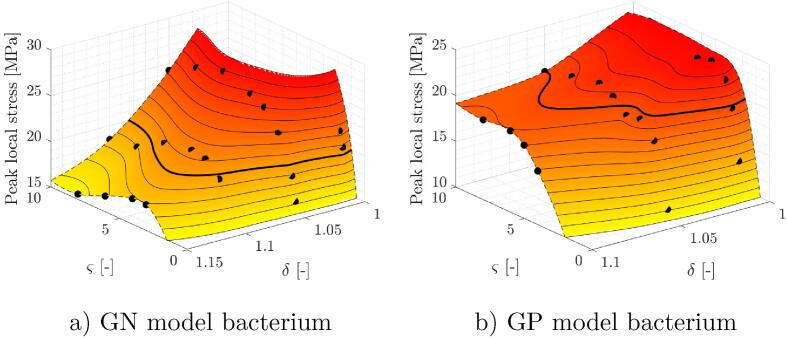
Peak values of stresses in the inner cell membrane across the considered parameter space δ-ς, as predicted by the numerical model for a) GN model organism and b) GP model organism. Both surfaces are obtained from the numerically evaluated cases (black dots) by thin plate spline interpolation and primarily serve for data visualization purposes. Solid black lines represent the interpolated peak stress contours with spacing of 1 MPa and both bold lines the estimated contours at the membrane poration threshold of 20 MPa (see Appendix A). Both surfaces are cut (dashed black line) along the edges of the considered δ-ς parameter space.

The presented results show that peak local stresses in both GN and GP bacteria can exceed poration thresholds of bacterial cell membranes. Additionally, the magnitude of calculated peak local stresses in the inner cell membrane shows high levels of variability on a relatively small fraction of the δ-ς parameter space. This can be seen in Fig. 9, where peak values of stresses in the inner cell membrane are given for both model organisms. Overall, peak stresses range between 11 and 27 MPa which could be the difference between cells being either unaffected or fragmented to pieces. Looking at the value distribution along both independent geometric parameters (horizontal axes), one can observe a monotonous increase of values with decreasing bubble-bacterium distance δ. This does not come as a surprise since our previous studies show similar trends for collapsing bubbles in vicinity of suspended spherical particles [14] and liposomes [15]. This is also a reason that presently a greater focus was given on bubble-bacterium size ratio ς. Although one could intuitively expect that larger bubbles would carry more damage potential, speculations could also be made about the extent of the cell membrane disruption - local poration versus complete destruction. The results of previous studies on single eukaryotic cells suggest that cell poration is not largely dependent on the bubble-cell size ratio, since experiments on cells much larger in comparison to cavitation bubbles ($R_{\mathrm{eq}}/R_{\mathrm{cell}}\approx 0.025$) [74] and vice versa ($R_{\max}/R_{\mathrm{cell}}\approx 6.5$) [75] show similar critical bubble-cell distances for membrane poration. Presently, we considered cases where ς assumed values between 0.75 and 10, which means that we consider bubbles of a similar size and larger bubbles than bacterial cells. We acknowledge that bubble-bacterial cell size ratio could also assume smaller values than 0.75. However then the bubbles are well into the territory of nanobubbles, where additional physiochemical effects come into play and thus other computational approaches might be more appropriate [76]. Coming back to Fig. 9, we can observe a steep increase in peak IM stress response with ς⩽4 to 6 for GN and ς⩽3 to 6 for GP model organism. From this one could conclude that larger bubbles carry a higher damage potential than bubbles of a similar size, when bacterial cells are considered. However, when bubble size surpasses a few multiples of cell size a non-monotonic trend emerges and the relation is not so clear. This can be explained by a significant elastic response of cells, different modes of peak stress distribution in IM, and also a possible presence of resonance. The latter was already observed for red blood cells, which were stretched up to five times their initial size as a result of nearby laser induced bubble expansion and subsequent collapse [77]. Again, at this point potential effects of resonance can only be speculated and this phenomenon should be more thoroughly addressed in the future. Additionally, ς is generally not limited to values below ten. We decided not to consider values beyond ten, since numerous simulation runs were already required to capture a non-monotonic peak cell response with 0.75⩽ς⩽10. Furthermore, with large bubbles (ς≫1) we come across additional limitations, as the required spatio-temporal resolution of simulations ceases to scale with ς due to a nearby bacterial cell. Nevertheless, the results show practically spherical bubble dynamics for ς⩾8, which could be considered in future research as a reasonable simplification to reduce the overall model complexity and the required computational resources.

**Fig. 10:**
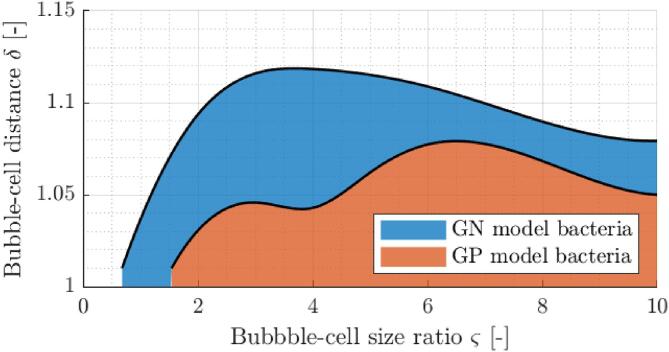
Estimated critical non-dimensional bubble-bacterium distances $\delta^{*}$ (solid black line) for poration of the inner cell membrane in relation to the bubble-bacterium size ratio ς. Regions in δ-ς parameter space where membrane poration threshold is exceeded are given for both Gram-negative (blue fill) and Gram-positive (orange fill) model cell.

As a result of non-monotonic peak stress response, a similar trend along ς also emerges when one considers critical non-dimensional distance for membrane poration $\delta^{*}$. The latter is estimated by interpolation between the obtained results and is represented by both bold contours at the membrane poration threshold of 20 MPa. For the sake of direct comparison between both model organisms, estimated critical distance and the corresponding regions in δ-ς parameter space where membrane poration threshold is exceeded are given in Fig. 10. One can notice that for ς⩽10 bacterial cell damage from a single bubble collapse is quite unlikely as cells have to be initially almost in contact with the bubble, irregardless of bubble size. Estimation of average poration distance across ς yields $\delta^{*}$ of 1.10 and 1.06 for GN and GP model cell, respectively. Furthermore, based on this we estimate that on average the IM poration and potential cell lysis of GN model bacteria is 12% more likely in comparison to the GP model bacteria during a single event of microbubble collapse:

(17) $${\left(\frac{\frac{1}{10-0.75}\int_{0.75}^{10}\delta_{\mathrm{GN}}^{*}d\varsigma }{\frac{1}{10-1.5}\int_{1.5}^{10}\delta_{\mathrm{GP}}^{*}d\varsigma }\right)}^{3}\approx {\left(\frac{1.10}{1.06}\right)}^{3}=1.12$$

At the first glance a difference of 12% might not seem as much, however when one considers a macro scale experiment, e.g. cavitating flow through a Venturi constriction, cavitation under an ultrasonic horn, etc., with many bubble collapse events the difference could add up and result in overall cleaning effect difference of a few orders of magnitude [6].

### 4.2 Mechanisms that Contribute to Bacteria Eradication by Cavitation Treatment

Numerous possible mechanisms that contribute to bacteria eradication by cavitation treatment have been speculated in the past [5], [4], [52], [13], ranging from mechanical (shear stresses, jets, pressure field variability, shock waves), to thermal and chemical. Similar mechanisms have been speculated in the case of other biological structures, such as liposomes [12], various eukaryotic cells [75], [78], [77], and viral pathogens [8]. Although the contribution of different mechanisms and their possible synergistic effects in various applications are still being explored [13], the most recent studies that address the use of hydrodynamic cavitation for wastewater treatment suggest that mechanical effects are the primary causes for inactivation of various biological structures [55], [9]. Similar speculations can be drawn from the results of a recent study [7], where E. *coli* bacteria were subjected to a low frequency ultrasound treatment and the rate ob bacterial cell sonolysis was primarily determined by the structural integrity of the peptidoglycan layer. Cells with removed peptidoglycan layer had sonolysis resistance equal to lipid vesicles and were extremely sensitive to sonolysis. The latter is in good agreement with the present study, where the GP model bacteria show higher resistance to bubble induced loads in comparison to the GN model cells, in spite the lack of an outer cell membrane. Similarly, when L. *pneumophila* (GN bacteria) and B. *subtilis* (GP bacteria) were subjected to hydrodynamic cavitation treatment the overall efficiency of cell removal was roughly one fold higher for L. *pneumophila* [6].

When one considers the contribution of various postulated mechanisms for bacteria eradication, the results of the present study clearly show that bacterial cell damage can be explained solely by mechanical effects in absence of thermal and chemical ones. Since the production of reactive oxygen species has presently not been modeled, we cannot draw any further conclusions regarding the possible synergistic effects of mechanical and chemical mechanisms. Additionally, we can identify thermal loads during a single microbubble collapse as a less likely mechanism of cell damage. However, further studies are needed to draw more concrete conclusions, as presently the bubble interior was modeled as ideal gas without the consideration of mass transfer mechanisms, which could affect the thickness of the developed thermal boundary layer. On the other hand, mechanical loads primarily include hydrodynamic loads that arise as a result of microstreaming and shock wave propagation through cells. The results further show that bubble-induced microstreaming is a predominating mechanism when more compliant cells are considered and when bubbles are similarly sized to cells. This is in line with previous studies that addressed bubble-liposome interaction and identified microstreaming as the primary driver of local liposome poration or even total vesicle destruction [10], [11], [15]. Shock waves on the other hand seem to have the greatest effect on cells that exhibit more rigid characteristics or in cases where bubbles are significantly larger than single bacterial cells. At this point it has to be stressed that the resulting response of bacterial cells to shock waves is also determined by the prior effects of microstreaming. The latter causes significant cell elongation during the phase of bubble contraction which is a prerequisite for a highly elastic response of cells to shock wave induced loads. This way we can identify microstreaming as the primary mechanism of bacterial cell damage, which in certain cases may be enhanced by the occurrence of shock waves during bubble collapse.

From this we can conclude that both geometric parameters of bubble-bacterium setup (distance, size ratio) and mechanical properties of bacterial cells determine the predominating cell damage mechanism. On the other hand, we weren’t able to find a clear relation between different modes of bubble collapse and cell damage mechanisms across the considered parameter space. We see this as an important factor, as generally damage potential of single bubbles is often attributed to the phenomenon of bubble jetting, where a characteristic high speed jet is developed towards the boundary acting as a jet driver. Although this might be well researched and accepted as one of the main damage mechanisms in the scope of material erosion when rigid boundaries are considered [50], the same cannot be said in the present case. Here presented results suggest that bubble jetting towards the cell is not a likely outcome of bubble-bacteria interaction, even when bubbles are initially almost in contact with the cell. Similar conclusions can be drawn from the results of other experimental and numerical studies that address interaction of bubbles with suspended eukaryotic cells [75], [77], [79], [80]. However, we do acknowledge that bubble jetting towards the cell could potentially still occur due to other jet drivers, such as shock waves, nearby bubbles, and rigid boundaries. In addition to jets, cell damage is often attributed to shear stresses or shear rates present in the flow field. Here a distinction between the source of shear stresses and their corresponding spatial scale has to be considered adequately. In the present paper, we show that cell damage can be expected even in a case of spherical bubble collapse where shear rate in the ambient liquid is effectively zero and only normal strain rates are present. It is true that a nearby cell does affect the local flow field development and can significantly change the resulting bubble dynamics. However, this is solely a result of bubble-cell interaction and not the underlying properties of the surrounding flow field which are determined by other factors, e.g., reactor geometry. In this manner, we see the rate of strain tensor or the corresponding strain rate magnitude as a more comprehensive metric.

## 5 Conclusions

In an attempt to further elucidate the process of cavitation-assisted water treatment, the present paper numerically addressed the interaction between a collapsing microbubble and a nearby compliant structure that mechanically and structurally resembles a bacterial cell. A fluid–structure interaction methodology was employed, where compressible multiphase flow was considered and bacterial cell wall was modeled as a multi-layered shell structure. Simulations were performed for two selected model structures, each resembling the main structural features of Gram-negative and Gram-positive bacterial cell envelopes. In addition, the contribution of two independent geometric parameters was investigated, namely the bubble-cell distance δ and their size ratio ς.

Three characteristic modes of bubble collapse were identified throughout the considered δ-ς parameter space. They range from the development of a weak and thin uniaxial jet away from the cell to spherical bubble collapses. This suggests that bubble jetting towards the cell is not a likely outcome of bubble-bacteria interaction in absence of other jet driving mechanisms, even when collapsing bubbles are initially almost in contact with the cell. The modes of cell deformation are found to be primarily governed by the rate of strain tensor of the ambient sink/source-type flow field. In addition, they vary significantly with the bubble-cell size ratio ς, as smaller bubbles have a more localized effect on nearby cells. The peak hydrodynamic forces and local shear stresses on the bacteria occur as a result of bubble collapse-induced shock wave propagation through cells. The former range from 2 to 7 μN for more compliant Gram-negative and from 10 to 22 μN for stiffer Gram-positive model cells, whereas the latter decrease with bubble-cell size ratio ς and vary between 0.5 and 5 MPa. Overall, four characteristic modes of spatial and temporal occurrence of peak local stresses in the inner cell membrane were identified. All modes occur near the time of bubble collapse or in the early phase of rebound. However, they are not restricted to the proximal region of the bacterial cell, as might be intuitively expected. The critical bubble-cell distance $\delta^{*}$ for membrane poration shows non-monotonic trends along ς and is estimated to be 1.10 and 1.06 for Gram-negative and Gram-positive model organism, respectively. The latter shows consistently higher resistance to the bubble-induced loads despite the absence of an outer cell membrane.

The results show that the local stresses arising from bubble-induced loads can exceed poration thresholds of cell membranes and that bacterial cell damage could be explained solely by mechanical effects in the absence of thermal and chemical ones. In addition, microstreaming is identified as the primary mechanical mechanism of bacterial cell damage, which in certain cases may be enhanced by the occurrence of shock waves during bubble collapse.

## CRediT authorship contribution statement

**Jure Zevnik:** Conceptualization, Formal analysis, Writing - original draft, Writing - review & editing. **Matevž Dular:** Conceptualization, Supervision, Project administration, Funding acquisition.

## Declaration of Competing Interest

The authors declare that they have no known competing financial interests or personal relationships that could have appeared to influence the work reported in this paper.

## Figures and Tables

**Fig. 11:**
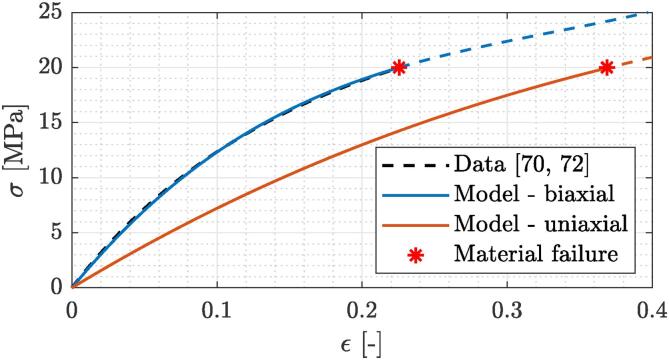
The considered material response of the inner cell membrane under biaxial (blue line) and uniaxial (orange line) loading as predicted by the Yeoh’s third order material model for incompressible hyperelastic materials. Black dashed line corresponds to the initial data obtained from Eq. (18) with $k_{A}^{0}=0.340$ N/m and $\tau_{0}=4$ nm [70], [72]. The expected onset of material failure is denoted by a red asterisk and the material response beyond that point is given in dashed style as the actual process of material failure is not presently included in the numerical model.

**Fig. 12:**
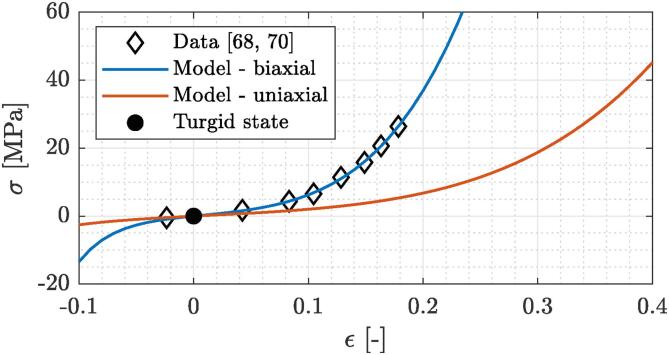
The considered material response of peptidoglycan layer under biaxial (blue line) and uniaxial (orange line) loading as predicted by the Yeoh’s third order material model for incompressible hyperelastic materials. Values are given in reference to the cell wall at normal turgid conditions (ε=0.225[68]). Diamond markers correspond to the source data [70] used for material model fitting.

**Fig. 13:**
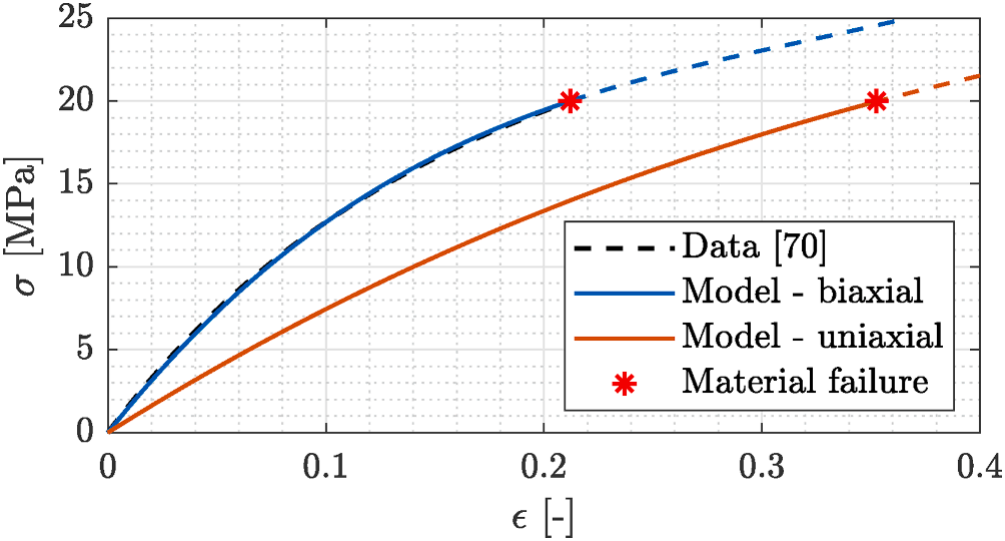
The considered material response of the outer cell membrane under biaxial (blue line) and uniaxial (orange line) loading as predicted by the Yeoh’s third order material model for incompressible hyperelastic materials. Dashed line corresponds to the initial data obtained from Eq. (18) with $k_{A}^{0}=0.525$ N/m and $\tau_{0}=6$ nm [70]. The expected onset of material failure is denoted by a red asterisk and the material response beyond that point is given in dashed style as the actual process of material failure is not presently included in the numerical model.

**Fig. 14:**
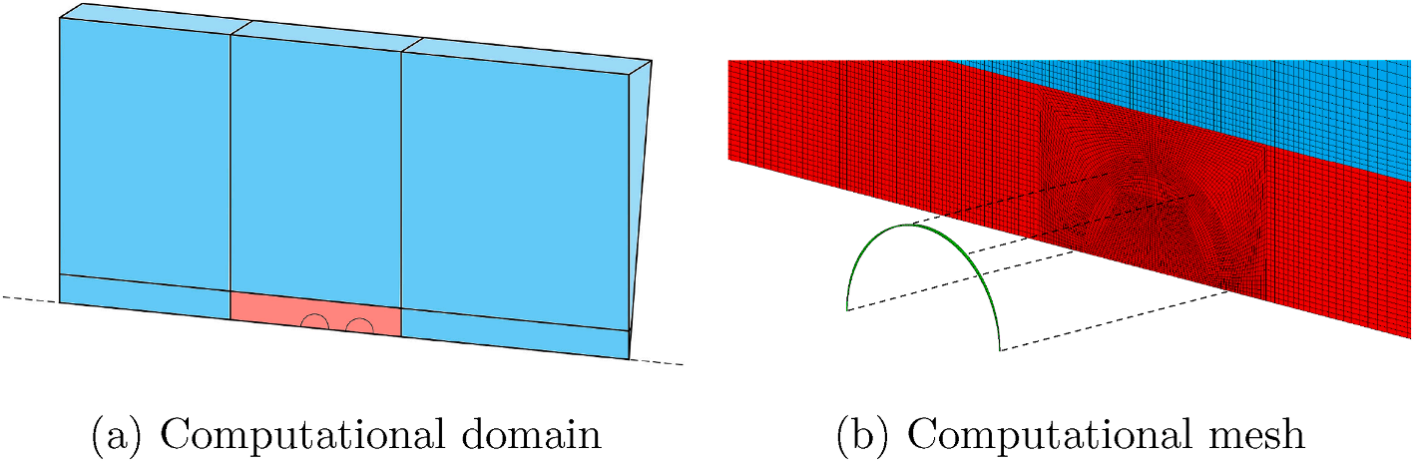
(a) A schematic representation of the utilized wedge geometry and the whole fluid computational domain. The wedge revolves around the axis of symmetry (dashed black line) with the thickness of one computational cell. The fluid domain is divided into two sections: a static mesh region (blue) and dynamic mesh region (red). Please note that the dimensions of the computational domain on are not directly proportional to the actual geometry for the sake of figure readability. (b) The computational mesh in direct vicinity of the FSI region showing mesh of the fluid domain (blue and red fill) and the structure domain (green fill). Sample mesh is given for a case with ς=1. For spatial reference, radius of the structure equals 1 μm. Please note that only a fraction of computational cells is shown and the structure domain is offset along the black dashed lines for the sake of visibility. Figure is taken from [15].

**Table 6:** Selected spatial and temporal resolution with respect to ς.

		Fluid domain		Structure domain	
ς [-]	Δt [ps]	Δx [nm]	$R_{0}/\Delta x$ [-]	$N_{\mathrm{cell}}$ [-]	Δx [nm]
0.75	2.5	4	188	864	3.6
1	3	5	200	690	4.6
1.5	3	6	250	576	5.5
2	4	8	250	432	7.3
3	5	10	300	345	9.1
4	6	13	308	267	11.8
5	7	16	313	432	7.3
6	8	19	316	348	9.0
8	10	25	320	300	10.5
10	12	30	333	270	11.6

## Appendix A Further Details regarding Modeling Bacterial Cells as Compliant Structures

Here we further describe and justify the utilized approach of modeling single bacterial cells as compliant structures. Each constituent of the bacterial cell wall - inner membrane (IM), peptidoglycan layer (PG), and outer membrane (OM), is modeled as incompressible hyperelastic solid with material characteristics based on the previous experimental and computational research. Bacterial cell interior is modeled as a Newtonian liquid.

### A.1 Bacterial Cell Wall

#### A.1.1 Inner Cell Membrane (IM)

From a mechanical standpoint, membrane stress σ in biaxial extension can be expressed as a function of linear strain ∊ [71]:

(18) $$\sigma =\frac{k_{A}^{0}\tau_{0}}{3}\left[1-{\left(\frac{1}{1+∊}\right)}^{6}\right],$$

where $k_{A}^{0}$ and $\tau_{0}$ are the area compressibility modulus and membrane thickness in the initial undeformed state (∊=0). In the present study, we consider the values of $k_{A}^{0}=0.340$ N/m and $\tau_{0}=4$ nm [70], [72]. The chosen value of $k_{A}^{0}$ is consistent with the fact that purely lipid bilayers exhibit area compressibility modulus in the order of ∼0.3 N/m when subjected to fast hyper-stretching [81], [82]. The IM is roughly 10% stiffer than the lipid bilayer as it also contains various membrane proteins, which are relatively incompressible in comparison to the lipid bilayer [70]. Here, it might be worth mentioning that experimental data of area compressibility modulus for lipid bilayers and biological membranes often show lower values in the range of ∼0.24 N/m [71]. This is due to the fact that experiments are often carried out at lower loading rates, where membranes also exhibit viscous properties in addition to the elastic response. For this reason biological membranes are generally classified as viscoelastic materials. However, material damping and viscoelasticity in the present case are not considered since viscous dissipation in the adjacent aqueous phase dominates the dynamic response of biomembranes to macroscopic shear deformations [83]. Additionally, the viscoelastic relaxation parameter for bacterial cell envelope is in the order of a second [84], which by several orders of magnitude exceeds the duration of herein considered phenomenon.

In the present case, we can expect an arbitrary stress state in the IM, which is the reason for the hyperelastic material model being employed in the actual numerical model. Through this we can account for the stress-softening characteristics of the IM. The stress–strain relation from Eq. (18) is used as initial data on which a Yeoh’s third order material model is fitted, as can be seen in Fig. 11. The corresponding material parameters obtained from the curve fitting process are: $C_{1}=1.33\times 10^{7}$ Pa, $C_{2}=-2.38\times 10^{6}$ Pa, and $C_{3}=6.28\times 10^{5}$ Pa.

Generally, the defect formation and subsequent membrane rupture is related to the strength of intramolecular forces within the bilayer. Membrane rupture can be expected at surface tension in the order of 80 mN/m [70], which at the initial membrane thickness of 4 nm translates to the stress of 20 MPa. This is also consistent with previous studies on other lipid membranes [85], [86], [87]. In this light we consider the same value of 20 MPa as the ultimate tensile strength of the IM on a temporal scale of 10–100 ns, which can be also understood as poration threshold. As the actual process of material failure is presently not included in the numerical model, we estimate the likelihood of material failure in retrospective by the phenomenological criterion of maximum stress where maximum principal stresses in the membrane are compared the poration threshold.

#### A.1.2 Peptidoglycan Layer (PG)

In the present study we consider PG as isotropic hyperelastic material that obeys the Yeoh’s thrid order material model. Through this we can account for a high degree of stress stiffening of the PG layer, which is crucial for the structural stability of bacterial cells. The inital data are taken from Hwang et al. [70], who reported the areal strains of a PG layer that arise at various surface tensions. As the authors report the values in reference to the undeformed state of the PG, we first transform the data to a reference turgid state at ${∊}_{A}=0.50$ (∊=0.225) [68] and then use it to fit the chosen material model (Fig. 12). The corresponding material parameters obtained from the curve fitting process are: $C_{1}=2.85\times 10^{6}$ Pa, $C_{2}=1.38\times 10^{7}$ Pa, and $C_{3}=2.61\times 10^{7}$ Pa. These yield the value of Young’s modulus in physiological turgid state to be in the order of 20 MPa, which is consistent with other studies on various Gram-positive and Gram-negative bacteria [69], [88], [89], [16], [90]. Additionally, the tangent modulus gradually increases with material stretching and already reaches a few hundred megapascal at the strains of ∊⩾0.35, where the IM is expected to start failing under uniaxial loading.

Besides stress stiffening, the PG can also exhibit anisotropic characteristics, which depends on the orientation of glycan strands and peptide bonds and varies across different bacterial species. This is also one of the determinants of bacterial cell shape. For example, PG layer in rod-shaped bacteria, such as E. *coli*, shows orthotropic behavior, with higher stiffness in the circumferential direction than the axial direction of the bacterial cell [69]. As already mentioned, in the present study we consider PG as isotropic hyperelastic material. Multiple reasons behind this exist. Firstly, we consider the most general shape of bacterial cells - spherical, which is associated with isotropic nature of PG. Secondly, in an attempt to consider PG as orthotropic hyperelastic material one notices a lack of available experimental data from the relevant literature. Although previous studies did address a similar topic [69], [89], the reported material parameters are not sufficient to form a full orthotropic hyperelastic material model. Additionally, the level of anisotropy is expected to vary across different bacterial strains and species and therefore remains a subject of the ongoing research.

#### A.1.3 Outer Cell Membrane (OM)

For the OM a similar approach to the case of IM is employed. Perhaps one of the main structural differences between the IM and OM is that the latter contains a lipopolysaccharide leaflet and presents as stiffer than the IM. Additionally, according to some research the OM can be also stiffer than the PG in normal turgid conditions [68]. In the present study, we consider the values of initial areal stiffness $k_{A}^{0}=0.525$ N/m and membrane thickness $\tau_{0}=6$ nm [70]. The corresponding material parameters obtained from the curve fitting process (Fig. 13) are: $C_{1}=1.38\times 10^{7}$ Pa, $C_{2}=-2.55\times 10^{6}$ Pa, and $C_{3}=7.21\times 10^{5}$ Pa. The same value of 20 MPa is assumed as the ultimate tensile strength of the OM on a temporal scale of 10–100 ns.

### A.2 Cell Interior

Cell interior is presently modeled as homogenous compressible Newtonian liquid. We acknowledge, that from a biological standpoint this is a vast oversimplification of the actual bacterial cytoplasm. However, from the mechanical point of view, the bacterial cell contents are mainly comprised of water (∼80 %) and do not consist of a traditional cytoskeleton, as can be found in eukaryotic cells. It is true that recent studies have shown, that some bacteria possess various proteins, e.g, FtsZ, MreB, crescentin, with a cytoskeleton-like function [91], which in fact does contribute to the cell’s structural integrity. However, these proteins are primarily considered to be important for maintaining non-spherical cell shapes and during the phase of cell division, both of which is not considered here.

Besides the main constituent, water, the bacterial cytoplasm contains enzymes, nutrients, gases, and cell structures such as ribosomes and plasmids, which is the reason for the average cell density in the order of 1100 kg/m^3^ [92]. Besides density, the viscosity of cytoplasm can also play an important role in the present case. Due to various constituents in addition to water, the bacterial cytoplasm exhibits varying effective viscosity. The latter depends on the size of a travelling object through it as larger objects are more affected by the intracellular contents on their path [93]. These findings, however, are primarily oriented towards a better understanding of intracellular molecular transport, which is not the topic of the present study. As the cell wall is generally considered to be the main determinant of bacterial cell response to the external mechanical loads, we decided to model cell interior as a viscous Newtonian fluid with density that matches the average bacterial cell density and other characteristics similar to water. Vastly increasing its viscosity would result in overly viscous behavior of cells on short time scales, which would result in unreasonably high shear loads exerted on the cell wall and its failure. This also would not be consistent with bacteria generally being highly resistant to various mechanical loads and behaving like elastic rods when subjected to transient hydrodynamic forces [16].

## Appendix B Further Details regarding Model Setup

### B.1 Computational Domain and Mesh Structure

All simulations are done in axial symmetry. The computational domain is resembled by a wedge geometry, which spans one computational cell in the direction around the axis of symmetry (Fig. 14a). The computational domain is split into two parts - fluid and structure domain, and a conformal mesh is used at their interface. Fluid domain boundaries are placed reasonably far away ($∼100R_{0}$) from the bubble-bacterium pair to minimize its influence on the considered phenomenon. Fluid domain is further split into two regions (Fig. 14 - a static mesh region (blue fill) and a dynamic mesh region (red fill). The latter is adapted according to the deformations of the structure domain during each iteration of the FSI procedure (see Fig. 2). A common deforming mesh method is employed, where edges between mesh nodes in a dynamic mesh region are represented as a network of linearly elastic springs. While this method might lack overall robustness, we have found during the preliminary simulations that it presently provides reasonable results with a significant reduction in computational load when compared to the Laplacian smoothing with distance-based diffusivity. A sample computational mesh is shown in Fig. 14b. Computational mesh of the fluid domain consists of orthogonal cells with a constant resolution in the region of bubble-cell interaction and is gradually coarsened towards the domain edges. Non-orthogonal quadrilateral cells are used in the direct vicinity and within the structure to transition between the spherical shell and the orthogonal cells in the rest of the computational domain. Each primary constituent of the cell wall (IM, PG, OM) is represented by a separate layer and together they form a layered spherical shell structure. The latter is defined with a surface through its mid-plane and is discretized with quadrilateral second order shell elements. Four in-plane and three through-thickness integration points are employed in each layer.

### B.2 Spatial and Temporal Resolution

Viscous and surface tension forces are scale-dependent and are known to have a cushioning effect on bubble collapse intensity. For this reason the finally employed spatio-temporal resolution (Table 6) was chosen based on preliminary simulations of unbounded microbubble collapse with $R_{0}$ between 0.75 and 10 μm. As the actual bubble-structure interaction might not necessarily result in spherical bubble dynamics, the finally obtained results were additionally controlled. From there we can conclude that in all the finally conducted FSI simulations the spatial resolution was ∼25 cells per minimum calculated bubble radius and that maximum local Courant numbers stayed below 0.6, even for the cases where bubble jetting occurred.

### B.3 Convergence Conditions

Between three and five coupling iterations per time step were performed on average to reach FSI data transfer convergence target of $10^{-3}$. Convergence criteria for the fluid dynamics solver were set with the values of scaled residuals for continuity and momentum equations to $10^{-6}$, and energy equation to $10^{-9}$. In addition, custom convergence criteria were also set to $10^{-6}$ for bubble radius, along with the integrals of pressure and shear stresses over the coupling interface, i.e., both sides of the cell wall. Convergence criteria in the structural solver were set as default vector norm checks ($L^{2}$ norm for force, moment, rotation and infinity norm for displacement) with the specified tolerance of $10^{-6}$.

## Appendix C Model Validation

Numerous practical and technical difficulties arise during experimental investigations on herein considered spatio-temporal scales. For this reason the authors can so far only offer various forms of indirect validation of the utilized methodology. Nevertheless, a substantial effort has been also put towards experimental observations of bubble-bacteria interaction by our research team. The preliminary results were already presented at multiple conferences [94], [95] and show that the required peak microbubble-induced hydrodynamic forces for cell lysis of E. *coli* amount to a couple of micronewtons, which compares very favorably with here obtained values for Gram-negative model bacterial cell (see Section 3.2). We see this as an important step towards validation of the employed modeling methodology of bacterial cells as compliant structures. Especially when considering the inherent experimental challenges and the fact that previously measured bacterial cell mechanical properties can vary up to a few orders of magnitude, even for the same bacterial species [69], [70], [68], [88], [96].

The presently employed fluid dynamics model was previously validated against the Gilmore’s model [97] for the case of an unbounded microbubble collapse [14]. A very good match between both models was observed until the first bubble collapse. Gilmore’s model predicted larger bubble rebound magnitude, which was attributed to different employed equations of state for bubble interior (adiabatic process considered in Gilmore’s model versus ideal gas model) and the observations of previous authors [98], [56], that the energy radiated into the surrounding liquid at the beginning of the rebound is not correctly predicted by the Gilmore’s model, which affects the magnitude of the rebound. Additionally, the model was validated against experimental results for laser-induced bubble dynamics in water and vegetable oil [58]. Multiple cases were considered: unbounded bubble in water, oil, and a bubble near a water–oil and oil–water interface. The results showed a good level of agreement until the second bubble collapse. The model was able to capture the phenomenon of bubble jetting towards the fluid interface with bubbles initiated in a lighter medium, and vice versa. Nevertheless, a temporal discrepancy between the experimental and numerical results for the initial phase of bubble growth was observed. The latter was attributed to a rather arbitrary initialization of the numerical simulations, which assumed an already developed flow field around the bubble, and did not account for the actual process of a laser-induced bubble formation. The employed model coupling framework [62] was previously shown to be capable of capturing complex multiphysics problems and has already been verified and validated in various engineering applications [99]. Furthermore, a similar FSI methodology was previously employed to addresses the interaction between a single cavitation microbubble and a nearby lipid vesicle of a similar size [15]. Results were not directly validated against experiments due to technical limitations at considered spatio-temporal scales (∼10 ns and ∼1μm). Nevertheless, the identified modes of vesicle deformation were qualitatively similar to the findings of previous experimental and analytical research. In addition, the estimated critical distance for vesicle poration and rupture showed a remarkably good agreement with previous experimental studies addressing single bubble-cell interaction.
