# Supplementary material for: Cavitation bubble interaction with compliant structures on a microscale: A contribution to the understanding of bacterial cell lysis by cavitation treatment
Source: Ultrason Sonochem. 2022 Jun 2;87:106053. doi: 10.1016/j.ultsonch.2022.106053 (PMC9190065; doi:10.1016/j.ultsonch.2022.106053)
Supplement: Supplementary data 1 [file mmc1.pdf]

# Supplemental Data
for
Cavitation Bubble Interaction with Compliant Structures on a Microscale: A Contribution to the Understanding of Bacterial Cell Lysis by Cavitation Treatment

Jure Zevnik[1,*] and Matevž Dular[1]

[1] *University of Ljubljana, Faculty of Mechanical Engineering, Aškerčeva cesta 6, Ljubljana, Slovenia*
[*] *Corresponding author, Email address: jure.zevnik@fs.uni-lj.si*

May 17, 2022

## List of Figures and Videos


S1 The actually considered value pairs $\delta - \varsigma$ for a) Gram-negative (GN) and b) Gram-positive (GP) model bacterial cell. . . . . 2
S2 Video of bubble and bacterial cell shape progression for a sample case that resembles bubble collapse mode J - weak jet away from the bacterium. . . . . 2
S3 Video of bubble and bacterial cell shape progression for a sample case that resembles bubble collapse mode T - transition between jetting and spherical bubbles. . . . . 3
S4 Video of bubble and bacterial cell shape progression for a sample case that resembles bubble collapse mode S - spherical collapse. . . . . 3
S5 Pressure and velocity field contours for a sample case that resembles bubble collapse mode T - transition between jetting and spherical bubbles. . . . . 4
S6 Pressure and velocity field contours for a sample case that resembles bubble collapse mode S - spherical collapse. . . . . 5


## List of Tables


S1 Temporal occurrence of peak cell elongation and maximum bubble size during rebound for GN model cell configurations. 5
S2 Temporal occurrence of peak cell elongation and maximum bubble size during rebound for GP model cell configurations. 5

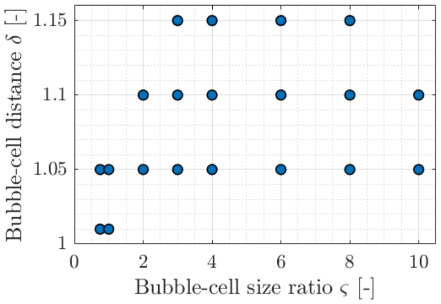


a) GN model bacterium

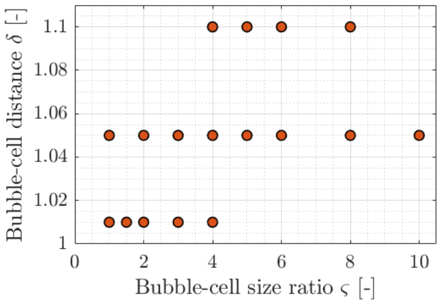


b) GP model bacterium

Figure S1: The actually considered value pairs $\delta - \varsigma$ for a) Gram-negative (GN) and b) Gram-positive (GP) model bacterial cell.

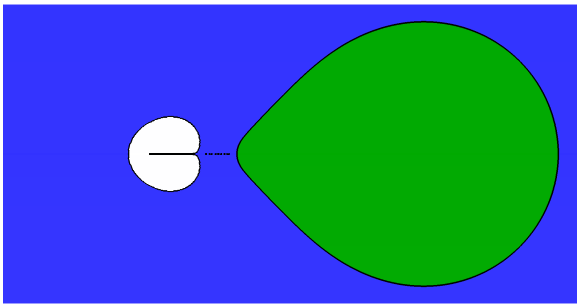

Figure S2: Video of bubble and bacterial cell shape progression for a sample case that resembles bubble collapse mode J - weak jet away from the bacterium: GP model cell, $\varsigma = 1$, $\delta = 1.01$. The corresponding bubble collapse time is $t_c = 9.98$ ns and the corresponding minimum equivalent bubble radius is $R_{eq} = 116$ nm. The peak bubble rebound is observed at $t/t_c = 1.53$, with $R_{eq}/R_0 = 0.52$. Bacterial cell is positioned on the right-hand side of the bubble and the shapes of both are marked by a solid black line.

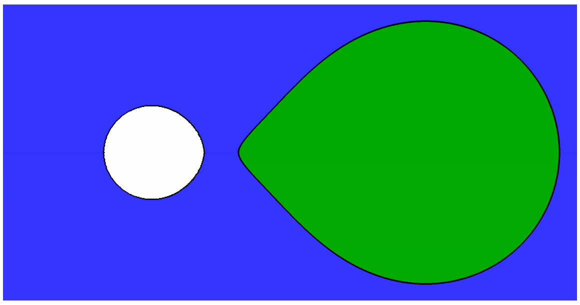

Figure S3: Video of bubble and bacterial cell shape progression for a sample case that resembles bubble collapse mode T - transition between jetting and spherical bubbles: GN model cell, $\varsigma = 1$, $\delta = 1.05$. The corresponding bubble collapse time is $t_c = 9.96$ ns and the corresponding minimum equivalent bubble radius is $R_{eq} = 115$ nm. The peak bubble rebound is observed at $t/t_c = 1.53$, with $R_{eq}/R_0 = 0.53$. Bacterial cell is positioned on the right-hand side of the bubble and the shapes of both are marked by a solid black line.

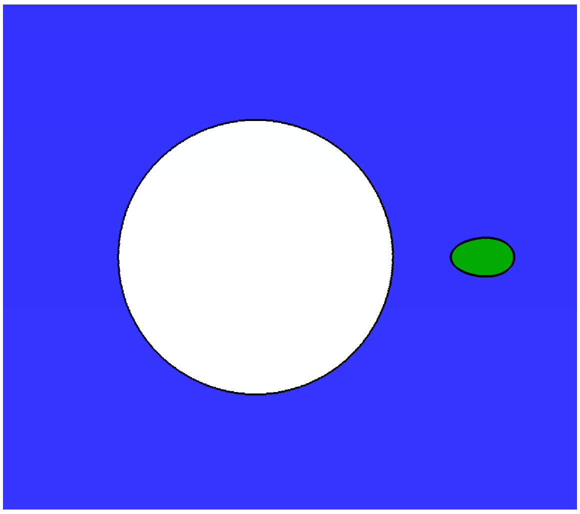

Figure S4: Video of bubble and bacterial cell shape progression for a sample case that resembles bubble collapse mode S - spherical collapse: GN model cell, $\varsigma = 10$, $\delta = 1.10$. The corresponding bubble collapse time is $t_c = 97.3$ ns and the corresponding minimum equivalent bubble radius is $R_{eq} = 0.77$ $\mu$m. The peak bubble rebound is observed at $t/t_c = 1.47$, with $R_{eq}/R_0 = 0.45$. Bacterial cell is positioned on the right-hand side of the bubble and the shapes of both are marked by a solid black line.

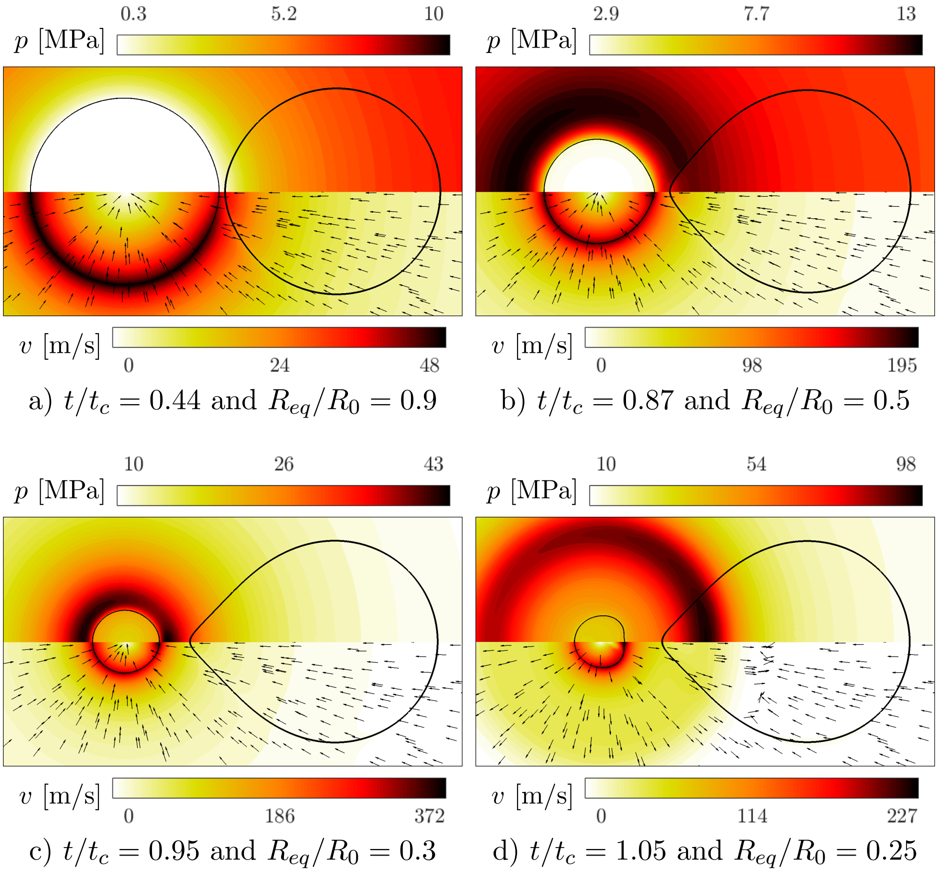


Figure S5: Pressure (upper half) and velocity (lower half) field contours for a sample case that resembles bubble collapse mode T - transition between jetting and spherical bubbles: GN model cell, $\varsigma = 1$, $\delta = 1.05$. The bubble collapse time for the selected case is $t_c = 9.96$ ns and the corresponding minimum equivalent bubble radius is $R_{eq} = 115$ nm. The peak bubble rebound is observed at $t/t_c = 1.53$, with $R_{eq}/R_0 = 0.53$. Bacterial cell is positioned on the right-hand side of the bubble and the shapes of both are marked by a solid black line. A corresponding video file showing bubble and bacterium shape progression is available in supplementary material (Video S3). The arrows in the lower half indicate only the direction of the velocity vector field.

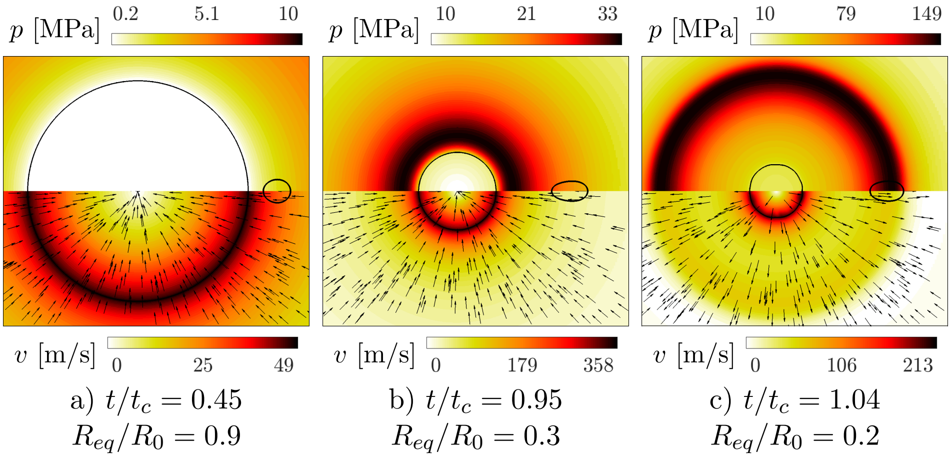


Figure S6: Pressure (upper half) and velocity (lower half) field contours for a sample case that resembles bubble collapse mode S - spherical collapse: GN model cell, $\varsigma = 10$, $\delta = 1.10$. The bubble collapse time for the selected case is $t_c = 97.3$ ns and the corresponding minimum equivalent bubble radius is $R_{eq} = 0.77$ $\mu$m. The peak bubble rebound is observed at $t/t_c = 1.47$, with $R_{eq}/R_0 = 0.45$ Bacterial cell is positioned on the right-hand side of the bubble and the shapes of both are marked by a solid black line. A corresponding video file showing bubble and bacterium shape progression is available in supplementary material (Video S4). The arrows in the lower half indicate only the direction of the velocity vector field.

| | | $t/t_c$ | |
|---|---|---|---|
| $\varsigma$ [-] | $t_c$ [ns] | Peak cell elongation | Peak bubble rebound |
| 2 | 19.66 | 1.007 | 1.504 |
| 4 | 39.07 | 0.998 | 1.483 |
| 8 | 77.89 | 0.986 | 1.469 |

Table S1: Temporal occurrence of peak cell elongation and maximum bubble size during rebound for GN model cell configurations shown in Fig. 5. The corresponding bubble collapse times $t_c$ are also included.

| | | $t/t_c$ | |
|---|---|---|---|
| $\varsigma$ [-] | $t_c$ [ns] | Peak cell elongation | Peak bubble rebound |
| 2 | 19.67 | 0.996 | 1.501 |
| 4 | 39.07 | 0.988 | 1.482 |
| 8 | 77.89 | 0.955 | 1.469 |

Table S2: Temporal occurrence of peak cell elongation and maximum bubble size during rebound for GP model cell configurations shown in Fig. 5. The corresponding bubble collapse times $t_c$ are also included.
